# Photoinhibiting via simultaneous photoabsorption and free-radical reaction for high-fidelity light-based bioprinting

**DOI:** 10.1038/s41467-023-38838-2

**Published:** 2023-05-27

**Authors:** Ning He, Xiaonan Wang, Liyang Shi, Jing Li, Lan Mo, Feng Chen, Yuting Huang, Hairong Liu, Xiaolong Zhu, Wei Zhu, Yiqi Mao, Xiaoxiao Han

**Affiliations:** 1 National Engineering Research Centre for High Efficiency Grinding, Hunan University, 410082 Changsha, China; 2 State Key Laboratory of Advanced Design and Manufacture for Vehicle Body, Hunan University, 410082 Changsha, China; 3 College of Biology, Hunan University, 410082 Changsha, China; 4 College of Food Science and Technology, Hunan Agricultural University, 410128 Changsha, China; 5 College of Material Science and Engineering, Hunan University, 410082 Changsha, China

**Keywords:** Biomaterials - cells, Biomaterials - cells, Polymers

## Abstract

Light-based 3D bioprinting is now employed widely to fabricate geometrically complex constructs for various biomedical applications. However, the inherent light scattering defect creates significant challenges in patterning dilute hydrogels to form high-fidelity structures with fine-scale features. Herein, we introduce a photoinhibiting approach that can effectively suppress the light scattering effect via a mechanism of simultaneous photoabsorption and free-radical reaction. This biocompatible approach significantly improves the printing resolution (~1.2 - ~2.1 pixels depending on swelling) and shape fidelity (geometric error less than 5%), while minimising the costly trial-and-error procedures. The capability in patterning 3D complex constructs using different hydrogels is demonstrated by manufacturing various scaffolds featuring intricate multi-sized channels and thin-walled networks. Importantly, cellularised gyroid scaffolds (HepG2) are fabricated successfully, exhibiting high cell proliferation and functionality. The strategy established in this study promotes the printability and operability of light-based 3D bioprinting systems, allowing numerous new applications for tissue engineering.

## Abstract (web summary)

‘Light-based bioprinting is employed in the fabrication of complex constructs but achieving high resolution remains challenging due to light scattering effects. Here, the authors develop a photoinhibiting additive which suppresses light scattering and demonstrate printing of functional scaffolds

## Introduction

Three-dimensional (3D) bioprinting technology, which allows cells and biomaterials to be patterned precisely, is a promising tool for fabricating highly sophisticated constructs for biomedical applications, such as tissue engineering, drug testing and surgical implants^1–6^. Among various 3D bioprinting modalities, digital light processing-based (DLP) printing has recently gained immense popularity due to its fine resolution (a few micrometres), rapid printing speed (seconds to minutes printing time) and predominant spatiotemporal controllability^1^. This technology converts liquid photocurable prepolymer into structured objects in a layer-wise fashion^7,8^ or via volumetric projection^9^ and has advanced bioprinting technology to create more elaborate structures, multi-vascular networks^10^, cell-laden scaffolds with multiscale channels^11^ and organ-on-a-chip systems^12^ for instance. In addition, DLP printers with high optical resolution (typically 1–50 μm) offer the possibility for fabricating 3D hydrogel constructs embedded with fine-scale features necessary to adequately recapitulate the complexities within the native tissue microenvironment and thus achieve appropriate biological behaviours in vitro^13–16^. However, recent studies^1,10,17–19^ have highlighted that the inherent physical light scattering defect can significantly deteriorate the printing resolution (defined as the printable minimal feature size relative to the printer’s optical resolution in pixel) of DLP and thus the capability in patterning architecturally-complex devices, hindering its application for more advanced tissue engineering.

Bioink for DLP printing typically consists of a photo-cross-linkable hydrogel (e.g., gelatin methacryloyl (GelMA)^20^, poly(ethylene glycol) diacrylate (PEGDA)^21^ and silk fibroin modified by glycidyl methacrylate (Sil-MA)^7^), a photoinitiator (e.g., lithium phenyl-2,4,6-trimethylbenzoylphosphinate (LAP)) and cells. GelMA, a natural protein biopolymer, is commonly used in 3D bioprinting due to its exceptional biocompatibility and cell-adhesive capability. However, it possesses poor mechanical performance and low viscosity, and a mixture of other materials (e.g., alginate/methylcellulose^22^ and PEGDA^23^) is usually implemented to enhance the printability of the bioink and the mechanical performance of printed objects. During the printing process, light scattering occurs at the interface where the refractive index is mismatched when light transmits within the bioink or the solidified polymer layers. This effect deviates propagating light to unilluminated regions (voids and channels, for instance), leading to out-of-target solidification and thus substantially deteriorating the printing resolution and geometric accuracy (referred to as fidelity) of the fabricated objects^1,10,17,24^. The hydrogel polymer can induce the scattering effect per se due to the formation of crosslinked networks that can increase the spatial inhomogeneity of hydrogels^19^. When cells, in the form of suspended particles, are encapsulated in the bioink, the scattering effect will be further enhanced because of the discrepancy in refractive index between the cytoplasm and external hydrogel environment^25,26^. It has been observed that particle-induced scattering can broaden the curing width by delivering more light radiation sideways, significantly deteriorating lateral resolution^27^. Another critical factor affecting pattern fidelity is the curing depth, which is vital in determining axial printing resolution. If the curing depth exceeds the optical depth of focus, the out-of-focus plane will polymerise, resulting in a deteriorated printing resolution and blockage of hollow structures^10,17,19^. Consequently, eliminating the undesired polymerisation caused by scattering and excessive light penetration is crucial for high-fidelity light-based 3D bioprinting.

A commonly used approach to resolve the issues above is doping the bioink with photoabsorbers, such as food dyes (e.g., tartrazine^28^ and Ponceau 4R^29^, anionic azo dye^30^ and 2-hydroxy-4-methoxy benzophenone-5-sulfonic acid (HMBS)^31^ that have enabled the reproduction of convoluted constructs embedded with multi-channels and vascular network^23,29,32^. However, the performance of the standard photoabsorbers is limited, deteriorating the capacity of the DLP printers they should possess^1^. Such an effect is more pronounced for platforms with a higher optical resolution^33^, particularly when cells are encapsulated^25^, creating significant challenges for patterning 3D cellularised constructs with fine-scale features (such as intricate micro-sized channels and thin-walled networks) that are of great importance to nutrient transportation and oxygen permeation. Notably, the existing photoabsorbing-based approach requires intensive trial-and-error efforts to optimise the printing parameters (e.g., light intensity) and bioink formulation (e.g., the concentration of each component) to achieve the desired pattern fidelity for a specific structure, tremendously hindering the practical use of DLP-based bioprinting technologies^17,34^. Such a trial-and-error approach is not only time-consuming and costly but also challenging to obtain the most optimal printing accuracy. Furthermore, previous studies have revealed that bioink with low-concentration hydrogels exhibits better cytocompatibility^35^; however, such a dilute nature imposes extra difficulties on the physical printing process. A fundamental issue of hydrogel photopatterning is how to simultaneously improve the printability for building complex constructs with high fidelity, simplify the operability for easy practical use and maintain appropriate biocompatibility for facilitating cell activities, a significant challenge in light-based 3D bioprinting technology.

Besides the photoinhibiting strategy, You et al. proposed a flashing photopolymerisation technique to suppress the scattering effect by minimising the light exposure time^19^. However, the cell-induced scattering effects cannot be resolved by using this method, making it inapplicable for fabricating cell-laden constructs with fine features. Recently, Guan et al. introduced a machine-learning approach to improve pattern fidelity by compensating for the scattering-induced geometric deviation when generating digital masks^25,36^. The intra-layer printing resolution can be enhanced using this method, yet performance for multi-layer fabrication of complex structures was not demonstrated. Therefore, a new biocompatible strategy that can effectively eliminate the light scattering effect is urgently required for light-based bioprinting.

Ideally, if the free radicals generated by light scattering can be removed or lowered below a threshold (e.g., through chemical reactions), the polymerisation of hydrogel prepolymers beyond designated regions can be prohibited. This paper presents a photoinhibiting approach that promotes the fabrication resolution and pattern fidelity of DLP bioprinting by addressing the light scattering problem, as well as minimising the trial-and-error optimisation of printing parameters. Unlike typical photoabsorbers, the photoinhibiting additive newly developed in this study can control the fundamental polymerisation process via simultaneous photoabsorption and free-radical reaction. The photoabsorption that functions as conventional photoabsorbers can prevent over-curing layers by absorbing excess light, thus improving the vertical printing resolution. The free-radical reaction can rapidly consume the free radicals generated by light scattering so that hydrogel monomers are starved at free radicals to form photo-crosslinked hydrogels in scattered regions, thereby impeding unwanted solidification. With this method, the printing resolution and pattern fidelity are significantly enhanced, and various complex 3D structures with multiscale features (particularly 3D cellularised scaffolds) can be fabricated all at once without re-tuning the printing parameters.

Firstly, based on the mechanism above, we synthesised a new photoinhibiting additive (curcumin-Na (Cur-Na)) that is water-soluble and cytocompatible. Secondly, PEG-GelMA bioinks added with various photoinhibiting additives were prepared for comparison, and the physico-chemical characterisations were performed to assess their printability and mechanical properties. The underlying mechanism of how Cur-Na mitigates the scattering effects was then elucidated by examining the polymerisation process theoretically and experimentally. Thirdly, the printing resolution and accuracy were investigated quantitatively for the bioinks to verify Cur-Na’s effectiveness for resolving the light scattering defect. Various architecturally-complex constructs were successfully fabricated using the Cur-Na bioink without re-optimising the printing parameters, demonstrating superb capability in patterning 3D functional cellularised scaffolds with fine-scale features (e.g., gyroid). Finally, the cytocompatibility of the Cur-Na bioink was confirmed through in vitro culturing experiments.

## Results and discussion

### Characterisation of Cur-Na

**Fig. 1:**
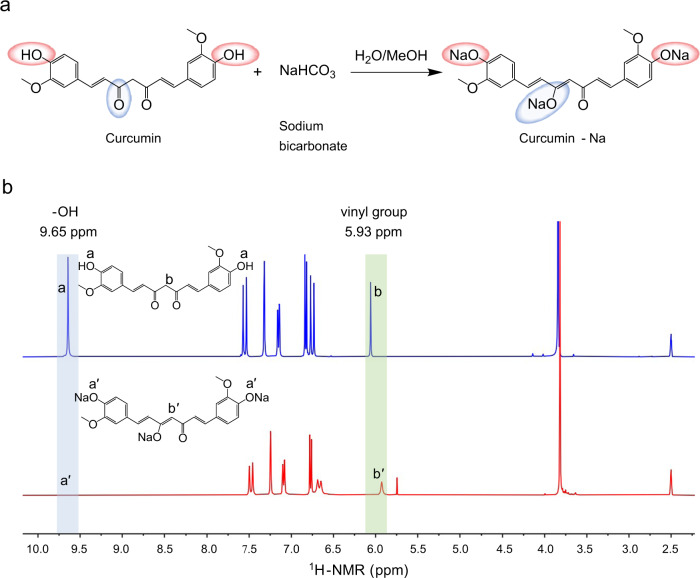
Synthesis of curcumin-Na. **a** Synthetic scheme of curcumin-Na (Cur-Na) using curcumin and sodium bicarbonate. The H^+^ of phenolic hydroxyl in curcumin molecular is substituted by Na^+^ (red ellipses), while the carbonyl bond in curcumin undergoes keto-enol tautomerism (blue ellipses). **b** ^1^H-NMR spectra of curcumin (upper blue) and Cur-Na (lower red), showing the representative changes of chemical shifts that occurred during the synthesis process. The blue-shaded area expresses the characteristic resonance of the phenolic hydroxyl group (*δ* = 9.65 ppm). The green shading is the alkene signal (*δ* = 5.93 ppm), indicating the formation of the C=C double bond.

**Fig. 2:**
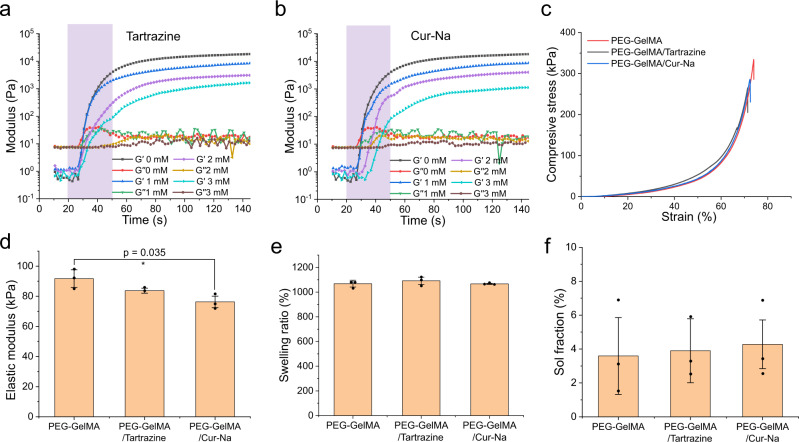
Physico-chemical characterisation of bioinks. Temporal evolution of storage modulus (*G'* reflecting the stiffness of materials) and loss modulus (*G''* reflecting the viscosity of materials) for bioink (PEG-GelMA/LAP) added with various concentrations of additives: **a** Tartrazine and **b** Cur-Na. The intersection between *G'* and *G''* is referred to as the gel point, while the gel time is defined as the beginning of exposure (20 s in this case) to the gel point. Bioink was exposed under 405 nm light with an intensity of 13 mW cm^−2^ for 30 s. Shaded areas express the exposure time. **c** Stress–strain curves of the hydrogels. *n* = 3 independent samples. **d** Elastic modulus of the three hydrogels at ~20% strains. *n* = 3 independent samples. Comparison of (**e**) swelling ratio and (**f**) sol fraction between three bioinks. *n* = 3 independent samples. Adding Cur-Na and tartrazine has a limited impact on the swelling ratio and sol fraction of the hydrogels. Data are presented as mean values ± standard deviation. The significant difference is de*t*ermined by two-tailed *t* test, *P < 0.05, **P < 0.01, and *P* > 0.05 (no significant difference).

To obtain a water-soluble reactive additive, we synthesised Cur-Na by chemically modifying curcumin using sodium bicarbonate, where the H^+^ of phenolic hydroxyl of curcumin molecular was substituted by Na^+^ (red ellipses in Fig. 1a). Meanwhile, the carbonyl bond in curcumin underwent keto-enol tautomerism due to the hydroxyl group in methanol, forming a new C=C double bond (blue ellipses in Fig. 1a). Such modifications were confirmed by proton nuclear magnetic resonance spectroscopy (^1^H-NMR). As shown in Fig. 1b, the characteristic resonance of the phenolic hydroxyl group (*δ* = 9.65 ppm) has disappeared by adding sodium to curcumin, indicating the substitution of H^+^. The formation of the C=C double bond was evidenced by the changes in the alkene signal (*δ* = 5.93 ppm), where a new signal peak representing the trisubstituted alkene was developed (Fig. 1b). The full spectrum of ^1^H-NMR and ^13^C-NMR are available in Supplementary Fig. 1 and Fig. 2. The existence of Na was determined by the ^23^Na-NMR spectrum (see Supplementary Fig. 3). The molecular weight measured by high-resolution mass spectrometry (HRMS) is 434, identical to that of the structural formula (see Supplementary Fig. 4 and Supplementary Table 1). The synthetic Cur-Na is a multifunctional additive that can be incorporated into bioink to improve printing quality. To verify its suitability for light-based 3D bioprinting, the light absorbance and water solubility were initially assessed experimentally. As shown in the absorbance spectra (Supplementary Fig. 5), Cur-Na demonstrated excellent light attenuation capability due to the absorbance peak (~425 nm) near 405 nm, a wavelength commonly used in light-based 3D bioprinting owing to its low detrimental effects on cells compared with UV light (e.g., 365 nm)^37,38^. Thus, Cur-Na can be used as a photoabsorber to effectively prevent over-curing layers, improving vertical printing resolution. In addition, Cur-Na has a water solubility of as large as 51 mg mL^−1^, making it well-suited for formulating water-containing bioink. Solution with low-concentration biomaterials (e.g., PEGDA and GelMA) and high water content is favourable for bioprinting because of the high bio-performance and good fluidity^34,35^. When Cur-Na monomers are dissolved in such a diluted bioink, the reactive functional groups (three C=C double bonds of alkene) can participate in the chain-growth reaction, offering extra controllability to the fundamental polymerisation process. Such a mechanism is of great importance in improving printing quality, which will be explained in detail in the section of photoinhibiting mechanism.

### Characterisation of bioink

Three pre-hydrogel formulations were developed for rheological studies, including PEG-GelMA/LAP (served as control), PEG-GelMA/LAP/tartrazine and PEG-GelMA/LAP/Cur-Na. For comparison, the photoabsorber tartrazine was adopted due to its biocompatibility, low toxicity and absorbance peak close to the light wavelength of the printer (405 nm). Both PEGDA and GelMA are typical hydrogels widely adopted for DLP printing^1,6^. Their crosslinking process is governed by free-radical chain polymerisation, and thus they are suitable candidates for evaluating the performance of the photoinhibiting method. A combination of PEGDA and GelMA was used because it preserves appropriate mechanical stability and cell adhesion, and has proven beneficial for functional recovery when serving as scaffolding materials^23,39,40^. Based on the working curves established for the bioink (see Supplementary Fig. 6), the layer thickness, light intensity, and exposure time were determined to be 100 μm, 13 mW cm^−2^ and 10 s, respectively. Such a set of parameters ensures the curing depth to be slightly larger than the layer thickness necessary to enhance the bonding of two neighbouring layers, while achieving the desired balance between printing speed and vertical resolution.

The photorheological characterisation was then carried out for the bioink in order to evaluate their printability and understand how Cur-Na affects the gelation kinetics of polymerisation. The temporal evolution of storage modulus (*G'* reflecting the stiffness of materials) and loss modulus (*G''* reflecting the viscosity of materials) for bioink added with various concentrations of tartrazine and Cur-Na are shown in Fig. 2a, b, where the intersection between *G'* and *G''* is generally referred to as gel point. Upon light exposure, the storage modulus rises rapidly at the early stage, followed by a moderate rate of change when the exposure time increases and stabilises ultimately. As indicated by the values of *G'* in Fig. 2a, b, increasing the concentration of both additives leads to a dose-dependent reduction in crosslinking density, meaning that excessive photoinhibiting additives can deteriorate the mechanical performance of the polymerised hydrogels. Grigoryan et al. demonstrated that PEGDA added with various amounts of tartrazine eventually reached an equivalent storage modulus by extending the exposure time beyond the reaction termination point, making the mechanical properties independent of the concentration of additives^10^. However, the extended irradiation time prolongs cell exposure to free radicals that can affect cell viability by causing damage to cell membranes and proteins^41,42^. The gel time, defined as the beginning time of exposure to the gel point (shaded area in Fig. 2a, b), is prolonged slightly (from 10.4 to 13.2 s) when the concentration of tartrazine increases to 3 mM, while it is doubled for Cur-Na (from 10.4 to 21.5 s), suggesting that the crosslinking rate is dependent on the concentration of additives and Cur-Na possesses a higher photoinhibiting efficiency compared with tartrazine. Such high efficiency is crucial for light-based bioprinting because a low concentration of Cur-Na is adequate to suppress the scattering effect while not reducing the stiffness significantly (see Fig. 2c, d). The discrepancy in both swelling ratio and sol fraction between the three types of bioinks is also negligible, and a low sol fraction value (<6%) was observed, suggesting that both Cur-Na and tartrazine have a limited impact on swelling and crosslinking kinetics (Fig. 2e, f). According to the analysis above, a concentration of 1 mM for both tartrazine and Cur-Na was chosen for further studies to ensure fast printing speed and appropriate mechanical performance of printed products.

### Photoinhibiting mechanism

**Fig. 3:**
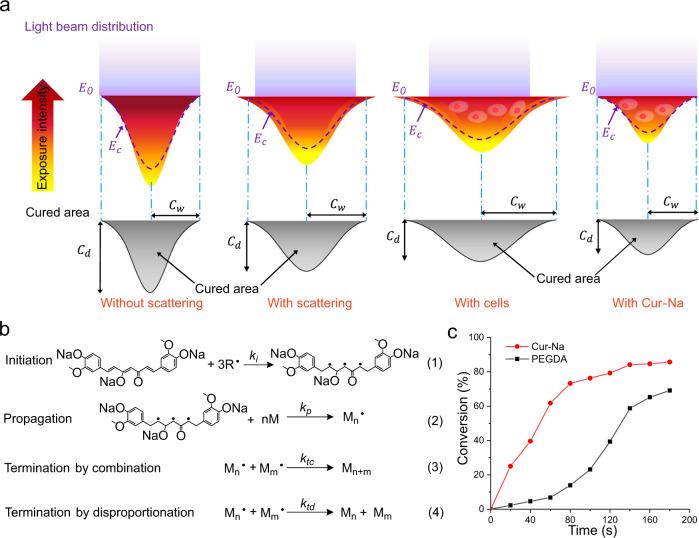
Mechanism of how Cur-Na suppresses the scattering effect. **a** Schematic diagram illustrating the light intensity profile of a Gaussian beam when the light penetrates a bioink and the resulting cured area for different situations, including without scattering, with scattering, bioink mixed with cell and bioink added with Cur-Na. *C*_*d*_ and *C*_*w*_ are the curing depth and curing width, respectively. *E*_0_ indicates the light intensity at the surface of the bioink, while *E*_*c*_ is the critical energy required to initiate polymerisation. **b** Kinetic scheme for free-radical chain-growth polymerisation of Cur-Na governed by three distinct stages: (1) initiation, (2) chain propagation and (3) & (4) termination. *k*_*i*_, *kp*, *k*_*tc*_ and *k*_*td*_ are rate constants of the four reactions. Each Cur-Na reacts with three radicals ($R\bullet$) to initiate polymer chain growth to form monomers (*M*). The radicalised monomers can react with other monomers and the polymer chain increases through propagation to generate polymer radicals (${M}_{n}\bullet$ and ${M}_{m}\bullet$). Polymers (*M*_*n*_ and *M*_*m*_) are formed when the chain propagation terminates via termination reactions. **c** The converted fraction of functional groups versus time during polymerisation for Cur-Na and PEGDA. The free radicals in scattered regions can be consumed by Cur-Na rapidly; hydrogel monomers are therefore starved at free radicals to form solidified polymers in scattered areas.

A light beam projected by a single micromirror in DLP is typically Gaussian, and its intensity decreases from the beam centre to the surrounding by obeying the Gaussian law^27,34^. When the incident Gaussian beam penetrates the bioink, it encounters exponential absorption governed by the Beer–Lambert law. As a result, the light intensity with a Gaussian profile is formed, resulting in a parabolic-like cured area depicted in Fig. 3a. The curing depth (*C*_*d*_) determines the vertical resolution and should be confined to the desired thickness to produce constructs of high resolution. Light scattering can alter the shape of the cured area by broadening the curing width (*C*_*w*_), substantially deteriorating the lateral resolution and resulting in poor pattern accuracy. In the presence of cells, the scattering effect is further amplified (see Fig. 3a).

Cur-Na, when dissolved in a bioink, can reduce light penetration and inhibit light scattering by interfering with the fundamental polymerisation process via simultaneous photoabsorption and free-radical reaction. As discussed previously, Cur-Na has an absorbance peak (~425 nm) near the wavelength of the light source (405 nm in this case) and can thus function as a typical photoabsorber to reduce the light penetration depth. In addition to this, Cur-Na can also suppress the scattering effect effectively by consuming the free radicals generated by scattered light. Mechanistically, the reactive functional groups of Cur-Na (three C=C double bonds of alkene) are capable of reacting with free radicals through a typical chain-growth polymerisation mechanism governed by three distinct steps, including initiation, propagation and termination (see Fig. 3b). Therefore, Cur-Na and hydrogels (e.g., PEGDA and GelMA) can react concurrently with free radicals in the presence of a photoinitiator and light. One concern was whether Cur-Na consumed free radicals more efficiently so that hydrogel monomers were starved at free radicals to form photo-crosslinked hydrogels in the scattered areas. To provide quantitative proof, the converted fraction of C=C double bonds versus time reflecting the polymerisation rate was measured for Cur-Na and PEGDA under identical polymerisation conditions. As illustrated in Fig. 3c, the polymerisation of Cur-Na occurs rapidly at an exceedingly high rate (the slope of the line) from the beginning to 80 s and decelerates when the converted C=C double bonds reach ~80%. In contrast, the polymerisation of PEGDA proceeds more slowly at the early stage, followed by a gradual acceleration after. The converted fraction of Cur-Na is continuously greater (~2–7 times) than that of PEGDA, signifying that Cur-Na owns a higher reactivity and a larger portion of free radicals was consumed by Cur-Na. As a result, solidified hydrogels cannot be produced by PEGDA in scattered regions due to insufficient free radicals. The high reactivity of Cur-Na can be ascribed mainly to 1) each Cur-Na molecular has three reactive C=C double bonds while it is two for PEGDA (see Fig. 3b1, 2) the molecular weight of Cur-Na (Mn = 436 Da) is much lower compared with that of PGEDA (Mn = 6000 Da), which can enhance the mobility of chains and thus accelerate the propagation process (Fig. 3b2) dramatically. Propagation generally dominates the overall polymerisation rate as all monomers (except one) are consumed during this step^43^. Another concern was whether, similar to PEGDA, the polymerisation reaction of Cur-Na can form crosslinked solidification in scattered areas, therefore deteriorating printing resolution. We have experimentally verified that the ultimate polymerised Cur-Na could not produce solidified polymers because of the insufficient crosslinked density resulting from the low molecular weight, even with a high concentration of Cur-Na (~5 wt%). Instead, a liquid state was observed after polymerisation, and the final polymerised products of Cur-Na can be washed out after printing.

On the one hand, once free radicals are generated in scattered areas, Cur-Na can prevent hydrogel monomers from being polymerised to form solidified hydrogels by competitively consuming free radicals at a significantly higher rate. On the other hand, unlike typical hydrogels, the reaction products of Cur-Na are liquid, thus avoiding generating unwanted solidification. Furthermore, a fixed concentration of Cur-Na can suppress the scattering effects over a wide range of light intensities due to its high reactivity, avoiding re-optimising printing parameters for fabricating different structures. Such a facile mechanism can effectively promote printing resolution and pattern fidelity.

### Printing resolution and fidelity

**Fig. 4:**
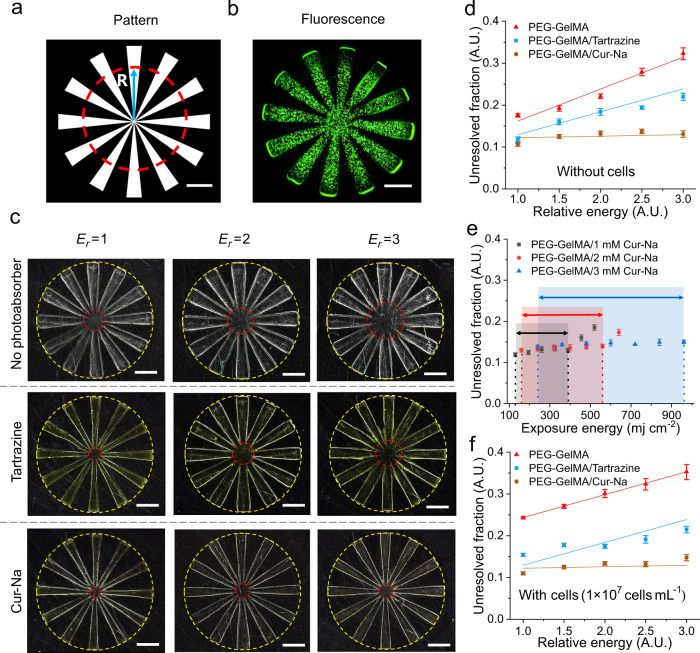
Lateral printing resolution analysis. **a** The spoke-like pattern employed to evaluate the printing resolution, showing the increased gap between adjacent spokes from the centre to the periphery. Resolution is characterised by the unresolved fraction, defined as the ratio between the unresolved diameter (red dashed circle) and the outer diameter. **b** Fluorescent image of the cell-laden structure. **c** Microscopic images of printed structures, showing the unresolved areas (red dashed circles) as a function of relative exposure energy for pure PEG-GelMA bioink (upper) and those added with tartrazine (middle) and Cur-Na (bottom). *E*_*r*_ is the relative energy defined as the ratio between the actual light energy and the unit exposure dose. Images b and c are representatives of *n* = 3 independent experiments. **d** Quantitative relation of unresolved fraction against exposure energy. A. U. standards for arbitrary units, a relative unit of measurement to represent the ratio of quantities. *n* = 3 independent sampl**e**s. **e** Printing window of Cur-Na showing the exposure energy range within which the resolution is maintained. The grey, red and blue-shaded areas indicate the window width of 1 mM, 2 mM and 3 mM Cur-Na, respectively. *n* = 3 independent samples. **f** Quantitative relation of unresolved fraction against exposure energy for cell-laden structures. *n* = 3 independent samples. Data are presented as mean ± standard deviation. All scale bars indicate 1 mm.

To demonstrate Cur-Na’s capability in improving printing resolution and pattern fidelity, we first evaluated the lateral resolution of printed objects quantitatively and compared the resolution differences between the three groups of bioink developed previously. A spoke-like pattern (see Fig. 4a), initially proposed by You et al.^19^, was adopted to quantify the printing resolution by calculating the unresolved fraction defined as the ratio between the unresolved diameter (marked as the red dashed circle in Fig. 4a) and the outer diameter. Given that DLP printing is a process in the nature of polymerising materials layer-upon-layer, it is, therefore, logical to investigate the resolution of a single layer prior to multi-layer fabrication. Spoke-like structures with a single layer of 100 μm were thus fabricated. Various light intensities were used to test the resolution sensitivity on light energy dose, which helps identify the tolerance window of printing parameters and whether re-optimisation is required for printing different structures.

Figure 4c displays the microscopic photographs of printed structures in which cells were not included, showing the unresolved areas (red dashed circles) as a function of relative exposure intensity for pure PEG-GelMA bioink and those added with tartrazine and Cur-Na. It can be observed that the pure PEG-GelMA bioink exhibits the greatest unresolved fraction, and the discrepancy in the size between the printed spokes and those designed (Fig. 4a) is noticeably large due primarily to the over-curing resulting from scattering. This underlines the fact that a simple structure cannot even be fabricated without doping a photoinhibiting additive, as seen in several previous studies^9,10,17,30^. It is also evident that the unresolved region is enlarged when light energy increases. When adding 1 mM tartrazine to the bioink, the resolution sensitivity on exposure intensity is still apparent despite the unresolved fraction for all energy doses being lowered. In the case of Cur-Na, the resolution is improved significantly, as indicated by the shrinking unresolved circles in the lower panel of Fig. 4c. The sensitivity to light intensity is minor, even though it is visually noticeable in the figure. To quantify the resolution difference, the relation between the unresolved fraction and relative energy is plotted in Fig. 4d. It can be seen that the resolution of the pure PEG-GelMA bioink is highly dependent on energy dosage, and the unresolved fraction is amplified from ~17% to as large as ~33% when the exposure intensity is tripled. A similar trend was observed for tartrazine, with reduced values that are ~12% and ~21% for *E*_*r*_ = 1 and *E*_*r*_ = 3, respectively. Unlike tartrazine, the unresolved fraction of Cur-Na remains at an almost constant value irrelevant to the exposure intensity, being as low as ~12%, suggesting that a fixed concentration of Cur-Na can deal with scattering with different intensities while preserving higher printing resolution. Figure 4e depicts the printing window that characterises the extent of exposure energy within which the printing resolution is maintained, serving as a quantitative guide for selecting the concentration of Cur-Na. For other photoabsorbers (e.g., Ponceau 4 R), a trend similar to tartrazine was observed due to their comparable absorbance peaks, being ~405 nm and ~500 nm for tartrazine and Ponceau 4R, respectively (see Supplementary Fig. 8).

When cells are encapsulated (see the fluorescent staining image in Fig. 4b), the scattering effect of the pure PEG-GelMA bioink is more pronounced, further increasing the unresolved areas and thus reducing the printing resolution (Fig. 4f and Supplementary Fig. 9). For tartrazine, when the exposure intensity that substantially governs the degree of scattering increases, the resolution is lowered due to enhanced scattering (Fig. 4f). A higher concentration of tartrazine might be required to compensate for the increased over-curing. However, it is at the expense of sacrificing the structure’s stiffness and printing time because there is a trade-off between printing resolution, printing efficiency and mechanical performance, mediated by the concentration of photoabsorber^1,17,20^. Purely increasing the amount of tartrazine or Ponceau 4R cannot improve the printing quality (see Supplementary Fig. 8c, d). Comparing the cell-free (Fig. 4d) and cell-laden (Fig. 4f) structures printed with Cur-Na, the influence of the cell-enhanced scattering on printing resolution is ignorable due to the scattering suppression mechanism of Cur-Na described previously. The results above reveal that Cur-Na can improve printing resolution better than a purely photoabsorbing-based method and that a fixed low concentration of Cur-Na is sufficient to suppress the scattering effect of various strengths, therefore simplifying the operational process. For a purely photoabsorbing-based method, the concentration of a photoabsorber (e.g., tartrazine) needs to be adjusted to match a unique scattering characteristic to achieve a better printing resolution^17^. However, the scattering characteristic varies dramatically along with changes in light intensity, formulation of bioink, exposure time, shape of structures and cell density^34^.

Matching the printing resolution with the optical resolution of printers is one of the greatest challenges for DLP printing because of the light scattering issue and the dilute nature of the hydrogels (swelling and low viscosity)^32^. The sensitivity of printing resolution to the concentration of hydrogels and various photoinhibitors was investigated to understand how light scattering and swelling impact the printing resolution (see Supplementary Fig. 10). The PEG-GelMA/Cur-Na hydrogel used in this study demonstrated a printing resolution of ~2.1 pixels, while a resolution (~1.2 pixels) of proximity to the printer’s optical resolution was achieved when the concentration of PEGDA was increased to 30% due to weakened swelling. The full printing capacity of DLP printers (1 pixel) is thus achievable using the method proposed in this study once the swelling of printing ink is confined entirely. For 30% PEGDA hydrogels, the resolution dropped from ~1.2 to ~2.7 pixels when replacing Cur-Na with standard photoabsorbers, although extensive parameter tuning was conducted. Such a change is due mainly to the scattering effect because the swelling ratio is almost identical, indicating that Cur-Na can suppress light scattering more effectively.

**Fig. 5:**
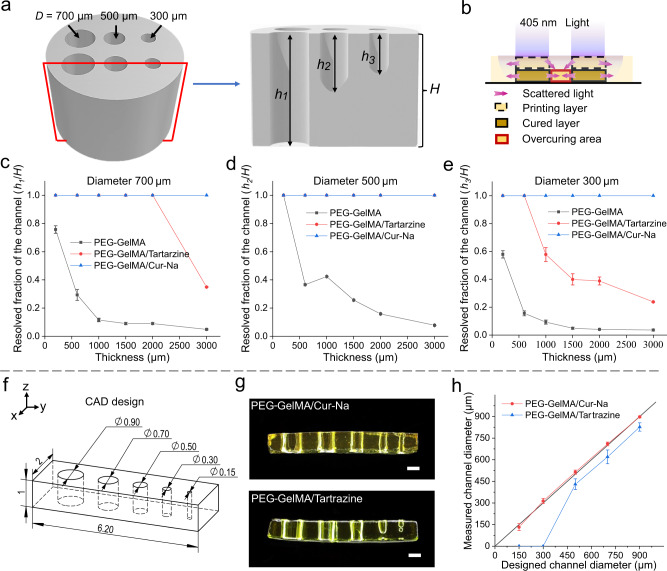
Hollow-channel printability analysis for multi-layer fabrication and printing error analysis for assessing high-fidelity capability. **a** 3D CAD design of a cylinder featuring open channels with various diameters (*D* = 300, 500 and 700 μm). *H* expresses the height of the cylinder while *h*_1_, *h*_2_ and *h*_3_ are the hollow fraction of the printed channels. The printing performance is characterised by the resolved fraction, defined as the ratio between the hollow section and the total length of the channel. **b** Schematic diagram demonstrating the scattering effect from consecutive layers and the resulting over-curing phenomenon. **c**–**e** illustrate the relation between the resolved fraction and the cylinder height for diameters of 700, 500 and 300 μm, respectively. *n* = 3 independent samples. **f** CAD design of the multi-channel structure with various diameters ($\varnothing$ = 900, 700, 500, 300 and 150 μm). **g** Samples printed using the PEG-GelMA/Cur-Na and the PEG-GelMA/Tartrazine bioink. The images are representatives of *n* = 3 independent experiments. Scale bars indicate 500 μm. **h** Comparison between the designed diameters of channels and those measured. The solid black line (*y* = *x*) indicates the perfect match between the designed and printed dimensions. Measured diameters were set to 0 once blockage occurred. *n* = 3 independent samples. Data are presented as mean ± standard deviation.

We then investigated how the scattering effect during multi-layer fabrication can be inhibited by Cur-Na, thus improving the printing quality of 3D structures. Biofabricated hollow structures are of particular importance for biomedical applications because they are essential for guiding tissue regeneration, forming vascular networks and accommodating cell attachment and growth^1,11,16^. Cylindrical samples featuring open channels with various diameters (300, 500 and 700 μm) were DLP-printed for multiple heights ranging from 0.2 mm to 3 mm (see Fig. 5a). The blockage of channels might occur during the printing process due to over-curing resulting mainly from scattering (see Fig. 5b and Supplementary Fig. 11). To account for such effect, the resolved fraction, defined as the ratio between the hollow section and the total length of the channel, was adopted to characterise the printing performance. A value of 1 indicates that the channel is hollow and perfusable, while 0 means complete blockage. Figure 5c–e illustrates the variation of resolved fraction as a function of the cylinder’s height for diameters 300, 500 and 700 μm, respectively. All the channels were fabricated successfully using the bioink added with Cur-Na, demonstrating superior capability for printing 3D channelled structures. In the case of tartrazine, the printing performance decreases gradually when the diameter becomes smaller or the height increases, showing strong dependence on the number of printing layers. For instance, the resolved fraction is lowered from 1 to ~0.2 when increasing the cylinder height from 600 to 3000 μm (see Fig. 5e). As expected, the performance is further weakened when a pure PEG-GelMA bioink is used. The results above reveal an important effect that light scattering can be superimposed during the consecutive printing process, leading to the undesired solidification being remarkably enhanced when printing large structures. We observed that the bioink situated in the channels became denser slowly as the printing process proceeded. A possible reason is that the trapped bioink is not refreshed promptly due to a lack of recirculation, leading to increased crosslinking density^37^. In addition, the polymerised solid layers are highly scattering compared with the initially transparent liquid bioink^19^, which can further contribute to the accumulation of scattering.

To demonstrate the high-fidelity capability of Cur-Na, printing error analysis was carried out by comparing the geometric discrepancy between designed and DLP-printed. Following the previously proposed methods^17,44^, a multi-channel structure with various channel diameters (900, 700, 500, 300 and 150 μm) was designed to evaluate the printing errors for bioink added with tartrazine and Cur-Na (see Fig. 5f). As shown in Fig. 5g, all the open channels were fabricated satisfactorily without blockage using the Cur-Na bioink, achieving the smallest channel of 150 μm in diameter. However, in the situation of doping the bioink with tartrazine, the 150 and 300 μm channels were blocked due to the undesired solidification caused by scattering (see Fig. 5g). Figure 5h compares the designed diameters and those measured, where the diameters of blocked channels were assumed to be zero. It can be seen that the dimensions of the channels printed using Cur-Na match very well with those designed, and the average error is less than ~5%, indicating high printing accuracy. For tartrazine, the measured diameters are narrowed by ~50 μm owing to the over-curing compared with those designed, although we carefully optimised the concentration of tartrazine. Huh et al. investigated the impact of the concentration of the most used photoabsorbers on the printing accuracy of DLP^17^. They demonstrated that a fixed concentration of photoabsorbers cannot effectively inhibit the scattering effect if the diameter of the channel is too small, leading to over-curing and blockage. In contrast, light is over-blocked when the diameter increases, resulting in a larger printed diameter than that designed. There is a transition from over-curing to insufficient polymerisation owing to the dynamic changes in scattering intensity. Consequently, the concentration of photoabsorbers should be matched precisely with the scattering characteristic of a structure to minimise printing errors, creating significant challenges for practical use. Unlike photoabsorbers, Cur-Na is self-adaptive to the dynamic changes of scattering characteristics, allowing various high-fidelity 3D constructs to be fabricated without re-optimising printing parameters, as further highlighted in the next section.

### Performance of printing 3D structures

**Fig. 6:**
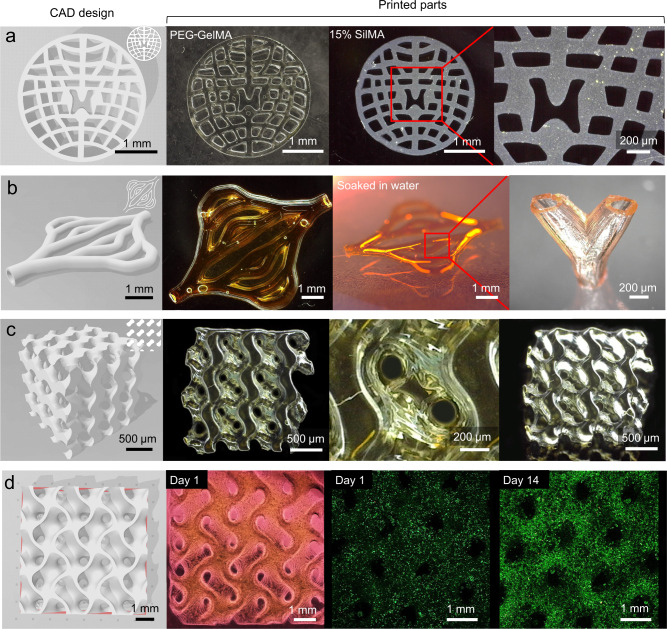
Demonstration of Cur-Na’s resolution and high fidelity capability in generating complex 3D structures commonly used in biomedical applications. **a** Spinal cord scaffold designed based on micro-CT scanning data. It features irregular channels and thin-walled networks that mimic the internal architecture of the spinal cord. The scaffolds were fabricated successfully using two types of hydrogels: PEG-GelMA (10%) and silk fibroin modified by glycidyl methacrylate (Sil-MA (15%)). **b** Vascular network with multiple branches, perfusable channels (200–800 μm) and thin walls (~100 μm). The perfusion was performed via injection with a coloured dye solution. **c** Gyroid scaffold with unique topological features (curved surface, high porosity and interconnectivity). **d** Printed gyroid scaffold encapsulated with HepG2 cells and cultured for 14 days (confocal images), showing high cell proliferation. Images a–d are representatives of *n* = 3 independent experiments.

To further demonstrate Cur-Na’s printability of complex 3D structures, convoluted scaffolds, including spinal cord scaffold, blood vessels and gyroid scaffold, were fabricated using the PEG-GelMA/Cur-Na bioink (Fig. 6). Neural scaffolds featuring parallel-aligned channels were usually adopted for biomimicking living neural constructs for spinal cord injury repair in previous studies^23,44–46^. A newly designed spinal cord scaffold was fabricated successfully using the Cur-Na bioink, showing irregular channels and thin-walled networks that can better mimic the internal architecture of the spinal cord (Fig. 6a and Supplementary Fig. 12a). The thickness of the thin-walled networks ranges from approximately 80 to 100 μm essential for effective exchange of metabolites and oxygen permeation^47^. Vascular networks with sizes of ~150–200 μm are necessary to establish adequate vascularisation for long-term cell activities; yet, it remains a considerable challenge for engineering tissue with vascular networks proximity to the size of capillary^34,48^. Based on our previously designed 3D vessel model for skin tissue regeneration^49,50^, a vascular network with multiple branches and perfusable channels (800–200 μm) was fabricated using the same bioink formulation (Fig. 6b), demonstrating high capability in generating vessels with vast length scales. The vascular wall (~100 μm) was well preserved, which differs from the previous DLP-printed hydrogel vascular networks^10,17,32^, where the channels were embedded within a bulk of hydrogel. The perfusion of the vascular network was confirmed via injection with a coloured dye solution (Fig. 6b and Supplementary Movie 1). Gyroid scaffolds made of quadruple junction points have shown promise in tissue scaffolding due to their unique topological features (e.g., curved surface, high porosity and interconnectivity) that yield optimal nutrient and waste diffusion for cell activities^29,50^. However, the architectural richness gives rise to the thin-walled structures that are difficult to manufacture. Most applications of 3D-printed gyroid scaffolds focus on hard tissue repair, and the printing materials (e.g., poly(ε-caprolactone) and hydroxyapatite) usually exhibit high stiffness^51,52^. Functional cellularised gyroid scaffolds engineered with hydrogels were, however, rarely reported. Huh et al. DLP printed a cell-laden gyroid scaffold at a scale of centimetres using the photoabsorbing-based method^17^. However, the cell viability and functionality were not demonstrated. In this study, gyroid scaffolds were successfully fabricated using the Cur-Na bioink, allowing the formation of ordered pores with a size of 150 μm (Fig. 6c). To demonstrate the performance for cell functionality, gyroid scaffolds encapsulated with HepG2 cells (1 × 10^7 ^mL^−1^) that are susceptive to light scattering were also fabricated successfully. High cell proliferation was observed after culturing for 14 days (Fig. 6d). Importantly, sphere formation was observed on day 7, and the protein expression of albumin increased rapidly after (see Supplementary Fig. 13), indicating enhanced hepatic functions. The cellularised gyroid scaffolds engineered with hydrogel offer numerous new applications for tissue engineering.

To demonstrate the feasibility of Cur-Na for other hydrogels, the spinal cord scaffold was fabricated using the Sil-MA^7^ bioink that has a printable window of 15–30% in concentration. Sil-MA bioink is opaque (strong light scattering) and the printed parts usually possess low compressive properties, thus more appropriate for exploring the performance boundary of Cur-Na. The lowest value of the printing window (15%) was employed in this study, although a concentration of 30% Sil-MA mixed with standard photoabsorber was used by Kim et al. to demonstrate its capability for patterning 3D complex structures^7^. Surprisingly, the scaffold was fabricated successfully using Cur-Na, precisely reproducing the designed fine-scale features (see Supplementary Fig. 12b). With standard photoabsorbers, the printing outcomes were, however, not ideal due mainly to intensive light scattering, demonstrating twisted thin-walled networks and blocked channels. Notably, the printed scaffold exhibited a low Young’s modulus (8–12 kPa depicted in Supplementary Fig. 12c, d) that is ~9× softer than the PEG-GelMA hydrogel used in this study, expanding the potential for constructing soft tissues such as liver and heart organs^53^.

### Cytocompatibility

**Fig. 7:**
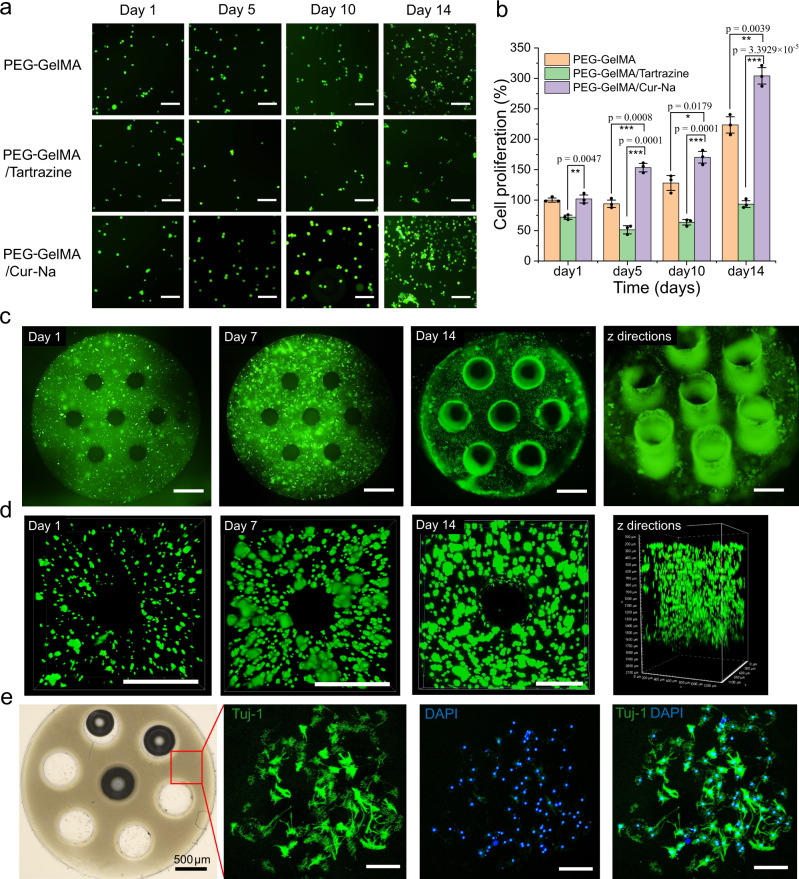
Cytocompatibility evaluation of Cur-Na based on in vitro culturing using PC-12 cells. **a** Live and dead assay stained images showing cell viability of encapsulated PC-12 cells for 14 days (live cells in green and dead cells in red). Scale bars indicate 100 μm. **b** CCK-8 assay; PC-12 cell proliferation in the cell-laden samples for 14 days. The PEG-GelMA/Cur-Na hydrogel exhibited the best cell proliferation. Each assay was conducted in triplicate. *n* = 3 independent samples. **c** Fluorescent images of the channelled scaffold (a diameter of 200 μm) fabricated using the PEG-GelMA/Cur-Na hydrogel, showing homogeneous cell distribution. The last image displays the cell growth along the channels. Scale bars indicate 500 μm. **d** Confocal images of the middle channel of the spinal cord scaffold on days 1, 7, and 14. Scale bars indicate 500 μm. **e** Fluorescence images showing differentiation and neurite formation of PC-12 cells (day 7) on PEG-GelMA/Cur-Na scaffold where the cells were seeded on the surface of the scaffold: Beta3-Tubulin (Tuj-1) and 4’,6-diaminyl-2-phenylindole (DAPI). White scale bars indicate 100 μm. Neurite formation was observed on day 7. Images (**c**–**e**) are representatives of *n* = 3 independent experiments. Data are presented as mean values ± standard deviation. The significant difference is determined by two-tailed *t* test, *P < 0.05, **P < 0.01, ***P < 0.001 and *P* > 0.05 (no significant difference).

To evaluate the cytocompatibility of Cur-Na for other cells, live/dead and CCK-8 assays for PC-12 cells were carried out for the three groups of bioink developed previously (Fig. 7a, b). It can be observed that the PEG-GelMA/Cur-Na hydrogel exhibited the uppermost cell viability, and the cell proliferation was approximately tripled after 14 days in culture, even higher than that of the pure PEG-GelMA bioink. The superior cytocompatibility is likely attributed to that Cur-Na is derived from curcumin, a natural phenolic antioxidant widely used for antioxidant, anti-inflammatory, antimicrobial, and so on^54^. Interestingly, the cell proliferation of the PEG-GelMA/Tartrazine hydrogel was not observed, even though tartrazine has been recognised as biocompatible and low-toxicity^10^.

To further demonstrate the suitability for tissue engineering applications, we fabricated a cell-laden scaffold featuring multiple channels (200 μm) using the Cur-Na bioink (Fig. 7c), followed by in vitro culture experiments to assess the cell growth behaviours. During the printing process, each layer with a thickness of 100 μm was printed within a relatively short period (10 s), which can effectively prevent the settling of cells. It can be seen that the cell distribution is homogeneous within the scaffold (Fig. 7c), which is key to enhancing cell communication and avoiding the cell aggregation effect. Microchannels with a diameter of 200 μm were patterned at a high concentration of cells (1–2 × 10^7 ^ mL^−1^), further highlighting Cur-Na’s printing performance for generating cell-laden 3D constructs. After 14 days in culture, the cells grew well along the wall of the channel (Fig. 7c, d), suggesting that the microchannels are capable of guiding cell growth, which can facilitate central nervous system regeneration^23,45^ and vascular network formation^49^. The structure remained stable after 14-day culturing; the size of channels was however amplified compared to that of day 1. It is likely due to the degradation phenomenon that can occur within hydrogel materials^8^. To verify whether Cur-Na and the generated hydrogel influence the differentiation behaviours of PC-12 cells, two differentiation tests were conducted by culturing PC-12 cells in the differentiation medium containing 1 mM Cur-Na (Supplementary Fig. 14) and by seeding the cells on the surface of the generated 3D channelled scaffold (Fig. 7e). After 7-day culturing, both differentiation and neurite formation were observed, demonstrating the potential for cell functionality studies.

In summary, we introduced an innovative photoinhibiting approach that can effectively suppress the inherent light scattering effect of light-based 3D printing platforms via a mechanism of simultaneous photoabsorption and free-radical reaction. We then synthesised a photoinhibiting additive (Cur-Na) based on such a mechanism and showcased its advantages by doping bioink with this additive, including (1) promoting the printing resolution and fidelity significantly. The full capacity of DLP printers (1 pixel) is achievable once the swelling of printing ink is confined; (2) simplifying the operating process by eliminating the re-optimisation procedures, which addresses the trial-and-error issue and has important practical and economic meaning; (3) superb performance in fabricating complex constructs featuring intricate micro-sized channels and thin-walled networks that are of great importance for nutrient transportation and oxygen permeation; (4) cell-laden gyroid scaffolds (HepG2 cells) that are susceptive to light scattering are fabricated successfully, exhibiting high cell proliferation and hepatic functions after 14-day culturing. (5) This mechanism is generic and applicable for other printing inks (e.g., Sil-MA) as long as the free-radical chain-growth polymerisation governs the printing process. This methodology offers a straightforward way to impart multiscale features into tissue constructs, which is vital for better mimicking the native tissue microenvironment. The strategy established in this study promotes the printability and operability of 3D bioprinting in patterning complex constructs with high fidelity, allowing for more advanced tissue engineering and regenerative medicine.

## Methods

### Curcumin-Na synthesis

Curcumin-Na was synthesised by chemically modifying curcumin using sodium bicarbonate. Specifically, 368 mg (1 mmol) curcumin (purity ≥98%, Aladdin) was dissolved in methyl alcohol (4 mL, purity ≥99.9%, Sigma-Aldrich) and deionised water (4 mL), and stirred until complete dissolution. Overall, 294 mg (3.5 mmol) NaHCO_3_ (purity ≥99%, Sigma-Aldrich) was then added to the solution to react with curcumin at ambient temperature. The mixture was dried by rotary evaporation and dissolved in 3 mL of anhydrous dichloromethane (purity ≥97%, Sigma-Aldrich). Filtering was performed subsequently to remove the insoluble matter. Finally, the solution was freeze-dried to obtain Cur-Na powder, and the yield was 82.9%. The Cur-Na powder was stored at −20 °C for further use. Note that methyl alcohol is toxic, and the synthetic process must be carried out under a ventilated condition.

### Characterisation (HRMS/NMR)

To determine the molecular weight of Cur-Na, the mass spectrum of Cur-Na was obtained by using high resolution mass spectrometer (HRMS) with a scanning range of 100–1000 m z^−1^. Cur-Na was manipulated and monitored under the electrospray ionisation (ESI) positive mode (Bruker Daltonics, Germany). The constituents of Cur-Na were identified by nuclear magnetic resonance (NMR) and the data were analysed using MestReNova14 software. ^1^H-NMR (400 MHz, DMSO-d_6_), ^13^C-NMR (101 MHz, DMSO-d_6_) and ^23^Na-NMR (159 MHz, DMSO-d_6_) spectra characterisation were performed via an NMR spectrometer (Bruker Avance III, Switzerland) using DMSO-d_6_ (purity ≥99.9%, Sigma-Aldrich) as the solvent. The samples were 0.7 mL DMSO-d_6_/6 mg Cur-Na, 0.9 mL DMSO-d_6_/25 mg Cur-Na and 0.9 mL DMSO-d_6_/20 mg Cur-Na for ^1^H-NMR, ^13^C-NMR and ^23^Na-NMR, respectively. The detailed peak list of NMR spectra and HMRS data are available in Supplementary Figs. 1–4 and Supplementary Table 1, respectively.

### Polymer and photoinitiator synthesis

Poly (ethylene glycol) diacrylate (6 kDa PEGDA) was synthesised following the protocol of Miller et al.^55^. Specifically, dry poly (ethylene glycol) (PEG; MW 6000, 81260, Sigma-Aldrich) was dissolved in anhydrous dichloromethane (purity ≥97%, Sigma-Aldrich). Triethylamine (2 molar over-dose to PEG, purity ≥99.5%, Sigma-Aldrich) and acryloyl chloride (4 molar over-dose to PEG, purity ≥97%, Sigma-Aldrich) were added into the mixture. The process was reacted overnight under argon gas. The yield was ~80%. The synthesised PEGDA was stored at −20 °C for further use. The synthetic process must be carried out under ventilated conditions.

Gelatin methacryloyl (GelMA) was synthesised following the protocol of Shirahama et al.^56^ with slight modifications. Specifically, 10 g gelatin (G1890-100G, Sigma-Aldrich) was added to PBS by stirring and the mixture was heated to 60 °C to obtain a gelatin solution. In total, 1.25 mL methacrylic anhydride (purity ≥ 94%, Macklin) was slowly added to the gelatin solution with continuous stirring. The mixture was reacted at 60 °C for 3 h under a pH from 8.0 to 9.0; the resulting products were dialysed in ddH_2_O for 3 days. The GelMA solution was frozen at −80 °C and lyophilised for 3 days. The yield was about 80%. The GelMA was stored at −20 °C for further use. The synthetic process must be carried out under ventilated conditions.

The photoinitiator lithium phenyl-2,4,6-trimethylbenzoylphosphinate (LAP) was synthesised following the method by Fairbanks et al.^57^. Specifically, dimethyl phenylphosphonite (0.186 mL, 1.17 mmol, purity ≥97%, Sigma-Aldrich) was added to an equivalent mole of 2,4,6-trimethylbenzoyl chloride (purity ≥97%, Sigma-Aldrich) and the mixture was then stirred overnight at room temperature under argon gas. The resultant products were mixed with excess lithium bromide (0.406 g, 4.68 mmol, purity ≥99%, Sigma-Aldrich) and 2-butanone (10 mL, purity ≥99%, Sigma-Aldrich). The mixture was subsequently heated to 50 °C for 20 min to ensure a complete reaction. Afterwards, the reaction products were rested for at least 4 h and cooled down to room temperature, followed by filtering the solution. The unreacted lithium bromide in the filtrate was then removed by adding the 2-butanone solvent, and the excess solvent was removed by rotary evaporation. The yield was about 80%. The LAP was stored at −20 °C for further use. The synthetic process must be carried out under ventilated conditions.

PEG-GelMA bioink composed of 6 wt% PEGDA (6 kDa), 4 wt% GelMA and 17 mM LAP was developed for DLP-based printing. Bioinks, including 15 wt% PEGDA (6 kDa)/25.5 mM LAP and 30 wt% PEGDA (6 kDa)/51 mM LAP, were developed for printing resolution tests. Sil-MA bioink composed of 15 wt% Sil-MA (EFL-Sil-MA-001, Yongqinquan Intelligent Equipment Co., Ltd., Suzhou, China) and 10 mM LAP was generated to verify the suitability of Cur-Na for other hydrogels.

### Physico-chemical properties

Rheological properties of bioink were obtained using a MCR 102 parallel plate rheometer (Anton Par, Austria) equipped with an optics attachment where a 405 nm projector was adopted as the light source. 6 wt% PEGDA, 4 wt% GelMA and 17 mM LAP added with various concentrations of tartrazine and Cur-Na were used as the bioink formulation. The bioink specimens were loaded onto the plate, and the top fixture was adjusted to obtain a gap size of 200 μm. The sweep was performed every 3 min with a frequency of 1 Hz and 10% strain. The 405 nm light irradiation was initiated at 20 s and lasted for 30 s with an intensity of 13 mW cm^−2^. The time-series data of storage modulus *G'* and loss modulus *G''* were then measured by RheoCompass software. A light exposure time of 30 s was selected because the resulting ∆*G'* log values (defined as the difference in the *G'* value of the crosslinked and uncrosslinked states) for PEG-GelMA/Cur-Na and PEG-GelMA/Tartrazine were ~78% and ~80% (see Fig. 2), respectively. Such values fall in the printable window (75–80% of ∆*G'* log values^34^), ensuring optimum crosslinking density.

Round-shaped samples (5 mm in diameter and 2 mm in depth) obtained by casting hydrogels into moulds were freeze-dried overnight to measure the dry weight (*m*_*dry t=*0_). The samples were then incubated overnight in adequate PBS at 37 °C to measure the wet weight (*m*_*swollen*_). Finally, the samples were further freezed-dried and weighed to obtain the dry weight (*m*_*dry*_). The swelling ratio can be calculated using the following equation^7^:

1 $$\%\,{swelling}\,{ratio}=\frac{{m}_{{swollen}}-{m}_{{dry}}}{{m}_{{dry}}}\times 100$$

The sol fraction of the hydrogel was calculated as follows^32^:

2 $$\%\,{sol}{fractio}n=\frac{{m}_{{dryt}=0}-{m}_{{dry}}}{{m}_{{dryt}=0}}\times 100$$

The compressive stress–strain measurements were carried out using a tensile-compressive tester (Shimadzu-AGS-X with 20 N sensor). The round-shaped samples are 5 mm in diameter and 2 mm in height. The hydrogels were incubated in PBS at 37 °C for 4 h prior to the test. The compression rate was 1 mm min^−1^, and the compressive modulus was obtained by calculating the rate of stress–strain curves at the strain value of ~20%. All tests of physico-chemical properties were repeated three times.

### Photopolymerisation kinetics

The polymerisation kinetics was characterised by measuring the conversion of C=C double bonds on real-time FT-IR spectroscopy (Nicolet iS10, Thermo Scientific, USA) equipped with a mercury cadmium telluride (MCT) detector. Samples (15 µL PEGDA or Curcumin-Na) were placed on an ATR crystal plate, and a 405 nm wavelength LED light was used for photopolymerising the materials. The light source was placed 3 cm above the sample, and the light intensity (13 mW cm^−2^) was calibrated and monitored using a Power Meter (PM16-401, Thorlabs, USA). The infrared absorbance spectra ranging from a wavenumber of 800–2000 cm^−1^ were recorded by Omnic software every 20 s for a total duration of 180 s (Supplementary Fig. 7), and the peak reflects the concentration of the double bonds. Each spectrum contains 16 scans with a resolution of 4 cm^−1^. The double bond conversion (*α*) was calculated as follows^18^:

3 $$\alpha=1-\frac{{A}_{t}}{{A}_{0}}$$

where, *A*_*t*_ and *A*_0_ represent the absorption area at an irradiation time point and before light irradiation, respectively. *A*_t_ and *A*_0_ were obtained by computing the integration of the absorbance peak over 940–1000 cm^−1^ (see Supplementary Fig. 7).

### 3D printing

All structures to be printed were designed using the Computer-Aided Design (CAD) 2021 software and sliced using the BMF 3D slicer software. These structures were fabricated by a projection micro stereolithography (PμSL) printer (NanoArch S140, BMF Material Technology Inc, Shenzhen, China). The PμSL printer was equipped with a 405 nm projector with a resolution of 10 μm in the *x–y* plane. A light intensity of 13 mW cm^−2^ was used throughout the study, except for cases of light-intensity sensitivity study. After printing, the 3D-printed objects were washed with PBS for at least 3 min. Microscopy images of the fabricated parts were obtained using the Dino-Lite microscope with the DinoCapture 2.0 software.

### Characterisation of the penetration depth

The relationship between the incident light intensity (*E*_0_) and curing depth (*C*_d_) of bioink was calculated from the following equation^1^:

4 $${C}_{d}={D}_{p}{{{{\mathrm{ln}}}}}\frac{{E}_{0}}{{E}_{c}}$$

where *E*_*c*_ and *D*_*p*_ are the critical energy required to initiate polymerisation and the penetration depth related to the light absorbance of bioink materials, respectively. *C*_*d*_ and *E*_0_ were measured from experiments. Four circular specimens were cured with different exposure doses *E*_0_ varying between 156 and 312 mJ cm^−2^ (see Supplementary Fig. 6a), and the cured thickness *C*_*d*_ was measured using Dino-Lite digital microscope (AM4815ZT, Dino-Lite, China). Finally, *E*_*c*_ and *D*_*p*_ were obtained by linearly fitting the logarithmic plotting of measured *C*_*d*_ against *E*_0_ by using the equation above (see Supplementary Fig. 6b, c).

### Characterisation of the printing resolution

A spoke-like pattern with sharp lines and an outer diameter of 5 mm (Fig. 4a) was designed and fabricated to evaluate the printing resolution of PEG-GelMA, PEG-GelMA/tartrazine and PEG-GelMA/Cur-Na. The printed structures were limited to a single layer thickness (100 μm) determined from the working curve in Supplementary Fig. 6. As the light energy to polymerise the three groups of bioink was different remarkably, the relative energy (*E*_*r*_) defined as the ratio between the actual light energy and the unit exposure dose was adopted for normalisation. The unit exposure dose was determined as the minimum light energy essential to solidify the spoke-like pattern properly. Five follow-up exposure doses *E*_*r*_ = [1, 1.5, 2, 2.5, 3] were used to polymerise the spoke-like structures, and the printing resolution was assessed by comparing the unresolved fraction of the fabricated objects. For printing cell-laden structures, PC-12 cells were encapsulated in the bioink with a cell concentration of 1 × 10^7 ^ mL^−1^. Fluorescence staining images were captured using Olympus inverted fluorescent microscope (IX-73, Olympus, Japan) with the Cellsens Standard software. The minimum printing resolution was tested via printing H letters designed with a linewidth of 1 pixel (10 μm). ImageJ was used to measure the printed structure’s dimensions (*n* = 3 per hydrogel).

### Hollow-channel printability analysis

Cylindrical structures featuring open channels with diameters of 300, 500, and 700 μm (Fig. 5a) were used to evaluate the printing performance of the bioink by measuring the extent of blockage of the patterned channels. The structures with heights of 200, 600, 1000, 1500, 2000, or 3000 μm were fabricated using the DLP printer with a layer thickness of 100 μm. The unblocked fraction of the channel was measured using Olympus inverted fluorescent microscope (IX-73, Olympus, Japan). All printed parts were washed in PBS for 3 min before image acquisition.

### Cells culture

PC-12 cells were initially induced with the nerve growth factor to obtain cells possessing neuronal characteristics. The PC-12 cells (from Procell Life Science&Technology Co., Ltd, China) were cultured in RPMI 1640 complete medium supplemented with 10% fetal bovine serum (Gibco Life Technologies, USA), 1% antibiotic solution (streptomycin, 100 μg mL^−1^, and penicillin, 100 units mL^−1^, Sigma-Aldrich Corp.). HepG2 cells (from Procell Life Science&Technology Co., Ltd, China) were cultured in high glucose Dulbecco’s modified Eagle’s medium (HG-DMEM) supplemented with 10% fetal bovine serum and 1% antibiotic solution. Culturing was performed in a humidified atmosphere incubator at 37 °C with 5% CO_2_ and 95% air.

### Cell viability and proliferation

Cells were added to PEG-GelMA, PEG-GelMA/Tartrazine and PEG-GelMA/Cur-Na bioinks, forming a solution with a cell density of 5 × 10^5 ^mL^−1^. A series of concentrations, including 2 × 10^5^, 5 × 10^5^ and 1 × 10^6^ mL^−1^, were tested. It was found that cells exhibited ideal viability and proliferation when a concentration of 5 × 10^5 ^mL^−1^ was selected. The printed cell-laden structures were cultured for 14 days in the above culture medium. Cytotoxicity was evaluated using a Live/dead assay kit (Beyotime Biotechnology, China) following the manufacturer’s instructions. Samples were visualised using a fluorescent microscope (IX-73, Olympus, Tokyo, Japan) after 30-min incubation at 37 °C and 5% CO_2_. The CCK-8 assays (Meilunbio, Dalian, China) were used to assess cell proliferation quantitatively (Thermo Scientific Multiskan FC), which was recorded by Skanlt RE 6.1.1 software.

### Characterisation of cytocompatibility of 3D cell-laden scaffolds

The cell-laden scaffolds were fabricated using the PμSL 3D printing system under sterile conditions. The bioink was filtered using a 0.22 μm filter. The PC-12 or HepG2 cells were added to the PEG-GelMA/Cur-Na bioink, and the cell density of the solution was 1–2 × 10^7 ^mL^−1^. The temperature of the bioink tank and the building platform were maintained at ~37 *°*C. The cell-laden 3D scaffolds were cultured in the culture medium for 1, 5, 10 and 14 days to observe cell proliferation. The culture medium was changed daily. Samples were imaged using a fluorescent microscope (IX-73, Olympus, Tokyo, Japan) and a confocal microscope (Ti-E + A1 MP, Nikon, Tokyo, Japan) after 30-min incubation at 37 *°*C and 5% CO_2_. Confocal images were obtained using the Nikon Ti-E + A1 MP microscope with the NIS-Elements Viewer 4.50 software.

### Albumin secretion

To evaluate the hepatic functions, the scaffolds culture supernatants were collected on days 1, 4, 7,10 and 14 and stored at −20 *°*C until use. The culture medium was changed daily, and at least three samples were used for each test. Albumin secretion was measured using the human albumin in vitro ELISA kit (SEKH-0081, Solarbio), and albumin concentration was calculated according to the experimental standard curve. Origin 2018 software was used for plotting.

### Cell differentiation

To investigate the PC-12 cell differentiation, PC-12 cells were cultured on HG-DMEM supplemented with 10% fetal bovine serum, 1% antibiotic solution and 50 ng mL^−1^ nerve growth factor (NGF). The differentiation medium was refreshed daily. After 7 days of culturing, the differentiation of PC-12 cells was observed by immunostaining. The samples were washed twice with PBS. Then the samples were fixed in 4% formaldehyde solution for 15 min and washed thrice with PBS. The samples were soaked in 0.2% Triton-X 100 for 10 min and then blocked in PBST solution with 5% FBS for 1 h, followed by incubating overnight with the primary antibody of Anti-beta III tubulin (Tuj-1, 1:500, ab18207, Abcam) at 4 *°*C. The samples were then repeatedly washed with PBS to remove unbound antibodies and incubated for 1 h with goat anti-rabbit 546 (1:500, a11305, Invitrogen). Finally, the samples were counterstained with 4’,6-diaminyl-2-phenylindole (DAPI, 1:1, GTX30920, GeneTex) for 15 min. Samples were imaged using a fluorescence microscope (IX-73, Olympus).

### Statistical analysis

The experimental results were present as mean values ± standard deviation (no less than three samples). The significant difference is determined by two-tailed *t* test, *P < 0.05, **P < 0.01, ***P < 0.001 and *P* > 0.05 (no significant difference). Significant differences between the means of parameters were calculated using the SPSS 17.0 software.

### Reporting summary

Further information on research design is available in the Nature Portfolio Reporting Summary linked to this article.

## Supplementary information

Supplementary Information

Description of Additional Supplementary Files

Supplementary Movie 1

Reporting Summary

## Data Availability

The data supporting the findings of this study are available in the paper and the Supplementary Information, and can be obtained upon request from the corresponding author fchen@hnu.edu.cn. Source data are provided together with this paper. Source data are provided with this paper.

## Source data

Source Data

## References

[1] Yu C (2020). Photopolymerizable biomaterials and light-based 3D printing strategies for biomedical applications. Chem. Rev..

[2] Mota C, Camarero-Espinosa S, Baker MB, Wieringa P, Moroni L (2020). Bioprinting: from tissue and organ development to in vitro models. Chem. Rev..

[3] Murphy SV, Atala A (2014). 3D bioprinting of tissues and organs. Nat. Biotechnol..

[4] Wang Y, Kankala RK, Ou C, Chen A, Yang Z (2021). Advances in hydrogel-based vascularised tissues for tissue repair and drug screening. Bioact. Mater..

[5] Ozbolat IT, Peng W, Ozbolat V (2016). Application areas of 3D bioprinting. Drug Discov. Today.

[6] Khoon SL (2020). Fundamentals and applications of photo-cross-linking in bioprinting. Chem. Rev..

[7] Kim SH (2018). Precisely printable and biocompatible silk fibroin bioink for digital light processing 3D printing. Nat. Commun..

[8] Ligon SC, Liska R, Stampfl J, Gurr M, Mulhaupt R (2017). Polymers for 3D printing and customized additive manufacturing. Chem. Rev..

[9] Brett EK (2019). Volumetric additive manufacturing via tomographic reconstruction. Science.

[10] Grigoryan B (2019). Multivascular networks and functional intravascular topologies within biocompatible hydrogels. Science.

[11] Xue D (2018). Projection-based 3D printing of cell patterning scaffolds with multiscale channels. ACS Appl. Mater. Interfaces.

[12] Yu F, Choudhury D (2019). Microfluidic bioprinting for organ-on-a-chip models. Drug Discov. Today.

[13] Park J, Wetzel I, Dreau D, Cho H (2018). 3D miniaturization of human organs for drug discovery. Adv. Healthc. Mater..

[14] Edmondson R, Broglie JJ, Adcock AF, Yang L (2014). Three-dimensional cell culture systems and their applications in drug discovery and cell-based biosensors. Assay Drug Dev. Technol.

[15] Antoni D, Burckel H, Josset E, Noel G (2015). Three-dimensional cell culture: a breakthrough in vivo. Int. J. Mol. Sci..

[16] Fang Y, Sun W, Zhang T, Xiong Z (2022). Recent advances on bioengineering approaches for fabrication of functional engineered cardiac pumps: a review. Biomaterials.

[17] Huh J (2021). Combinations of photoinitiator and UV absorber for cell-based digital light processing (DLP) bioprinting. Biofabrication.

[18] Zhao X (2021). Efficient 3D printing via photooxidation of ketocoumarin based photopolymerisation. Nat. Commun..

[19] You S, Wang P, Wang P, Hwang HH, Chen S (2019). High-fidelity 3D printing using flashing photopolymerization. Addit. Manuf..

[20] Pepelanova I, Kruppa K, Scheper T, Lavrentieva A (2018). Gelatin-methacryloyl (GelMA) hydrogels with defined degree of functionalization as a versatile toolkit for 3D cell culture and extrusion bioprinting. Bioengineering.

[21] Zhu W (2018). Rapid continuous 3D printing of customisable peripheral nerve guidance conduits. Mater. Today.

[22] Li H, Tan YJ, Kiran R, Tor SB, Zhou K (2021). Submerged and non-submerged 3D bioprinting approaches for the fabrication of complex structures with the hydrogel pair GelMA and alginate/methylcellulose. Addit. Manuf..

[23] Koffler J (2019). Biomimetic 3D-printed scaffolds for spinal cord injury repair. Nat. Med..

[24] Melchels FP, Feijen J, Grijpma DW (2010). A review on stereolithography and its applications in biomedical engineering. Biomaterials.

[25] Guan J (2021). Compensating the cell-induced light scattering effect in light-based bioprinting using deep learning. Biofabrication.

[26] Ferrage L, Bertrand G, Lenormand P, Grossin D, Ben-Nissan B (2016). A review of the additive manufacturing (3DP) of bioceramics: alumina, zirconia (PSZ) and hydroxyapatite. J. Aust. Ceram. Soc..

[27] Zakeri S, Vippola M, Levänen E (2020). A comprehensive review of the photopolymerisation of ceramic resins used in stereolithography. Addit. Manuf..

[28] Stevens LJ, Burgess JR, Stochelski MA, Kuczek T (2015). Amounts of artificial food dyes and added sugars in foods and sweets commonly consumed by children. Clin. Pediatr..

[29] Lim KS (2018). Bio-resin for high resolution lithography-based biofabrication of complex cell-laden constructs. Biofabrication.

[30] Simon U, Dimartino S (2019). Direct 3D printing of monolithic ion exchange adsorbers. J. Chromatogr. A.

[31] Ouyang X (2017). Optical micro-printing of cellular-scale microscaffold arrays for 3D cell culture. Sci. Rep..

[32] Levato R (2021). High-resolution lithographic biofabrication of hydrogels with complex microchannels from low-temperature-soluble gelatin bioresins. Mater. Today Bio.

[33] Raman R (2016). High-resolution projection microstereolithography for patterning of neovasculature. Adv. Healthc. Mater..

[34] Yu K (2021). Printability during projection-based 3D bioprinting. Bioact. Mater..

[35] Yin J, Yan M, Wang Y, Fu J, Suo H (2018). 3D bioprinting of low-concentration cell-laden gelatin methacrylate (GelMA) bioinks with a two-step cross-linking strategy. ACS Appl. Mater. Interfaces.

[36] You S (2020). Mitigating scattering effects in light-based three-dimensional printing using machine learning. J. Manuf. Sci. Eng.

[37] Wang Z (2018). A novel, well-resolved direct laser bioprinting system for rapid cell encapsulation and microwell fabrication. Adv. Healthc. Mater..

[38] Xie M (2019). Electro-assisted bioprinting of low-concentration GelMA microdroplets. Small.

[39] Cha C (2014). Structural reinforcement of cell-laden hydrogels with microfabricated three dimensional scaffolds. Biomater. Sci..

[40] Chen YC (2012). Functional human vascular network generated in photocrosslinkable gelatin methacrylate hydrogels. Adv. Funct. Mater..

[41] Choi JR, Yong KW, Choi JY, Cowie AC (2019). Recent advances in photo-crosslinkable hydrogels for biomedical applications. BioTechniques.

[42] Khoshakhlagh P, Bowser DA, Brown JQ, Moore MJ (2019). Comparison of visible and UVA phototoxicity in neural culture systems micropatterned with digital projection photolithography. J. Biomed. Mater. Res. A.

[43] Ravve, A. *Principles of Polymer Chemistry* (Springer, 2012).

[44] Li Y (2019). High-fidelity and high-efficiency additive manufacturing using tunable pre-curing digital light processing. Addit. Manuf..

[45] Liu X (2021). 3D bioprinted neural tissue constructs for spinal cord injury repair. Biomaterials.

[46] Li P, Xu Y, Cao Y, Wu T (2020). 3D digital anatomic angioarchitecture of the rat spinal cord: a synchrotron radiation micro-CT study. Front. Neuroanat..

[47] Fang Y (2020). Optimizing bifurcated channels within an anisotropic scaffold for engineering vascularized oriented tissues. Adv. Healthc Mater..

[48] Zhu W (2017). Direct 3D bioprinting of prevascularized tissue constructs with complex microarchitecture. Biomaterials.

[49] Han, X. et al. Optimized vascular network by stereolithography for tissue engineered skin. *Int. J. Bioprint*. **4**, 134 10.18063/IJB.v4i2.134 (2018). 10.18063/IJB.v4i2.134 PMC7582000 33102915

[50] Han X, Bibb R, Harris R (2016). Engineering design of artificial vascular junctions for 3D printing. Biofabrication.

[51] Feng J, Fu J, Yao X, He Y (2022). Triply periodic minimal surface (TPMS) porous structures: from multiscale design, precise additive manufacturing to multidisciplinary applications. Int. J. Extrem. Manuf..

[52] Zhang Q (2022). High‐strength hydroxyapatite scaffolds with minimal surface macrostructures for load‐bearing bone regeneration. Adv. Funct. Mater..

[53] Jung M, Ghamrawi S, Du EY, Gooding JJ, Kavallaris M (2022). Advances in 3D bioprinting for cancer biology and precision medicine: from matrix design to application. Adv. Healthc. Mater..

[54] Germain, L., Fuentes, C. A., van Vuure, A. W., des Rieux, A. & Dupont-Gillain, C. 3D-printed biodegradable gyroid scaffolds for tissue engineering applications. *Mater. Design* **151**, 113–122 (2018).

[55] Miller JS (2010). Bioactive hydrogels made from step-growth derived PEG-peptide macromers. Biomaterials.

[56] Shirahama H, Lee BH, Tan LP, Cho N-J (2016). Precise tuning of facile One-pot gelatin methacryloyl (GelMA) synthesis. Sci. Rep..

[57] Fairbanks BD, Schwartz MP, Bowman CN, Anseth KS (2009). Photoinitiated polymerisation of PEG-diacrylate with lithium phenyl-2,4,6-trimethylbenzoylphosphinate: polymerisation rate and cytocompatibility. Biomaterials.

