# Supplementary material for: Photoinhibiting via simultaneous photoabsorption and free-radical reaction for high-fidelity light-based bioprinting
Source: Nat Commun. 2023 May 27;14:3063. doi: 10.1038/s41467-023-38838-2 (PMC10224992; doi:10.1038/s41467-023-38838-2)
Supplement: Supplementary file 1 — Supplementary Information [file 41467_2023_38838_MOESM1_ESM.pdf]

SUPPLEMENTARY INFORMATION

Photoinhibiting via simultaneous photoabsorption and free-radical reaction for high-fidelity light-based bioprinting

Ning He[1,2], Xiaonan Wang[3], Liyang Shi[3], Jing Li[1], Lan Mo[4], Feng Chen[1*], Yuting Huang[5], Hairong Liu[5], Xiaolong Zhu[1], Wei Zhu[1], Yiqi Mao[1], Xiaoxiao Han[1,2*]

[1] National Engineering Research Centre for High Efficiency Grinding, Hunan University, 410082, China

[2] State Key Laboratory of Advanced Design and Manufacture for Vehicle Body, Hunan University, Changsha, 410082, China

[3] College of Biology, Hunan University, Changsha 410082, China

[4] College of Food Science and Technology, Hunan Agricultural University, Changsha, 410128, China

[5] College of Material Science and Engineering, Hunan University, Changsha, 410082, China

* Corresponding author email addresses: fchen@hnu.edu.cn; xiaoxiaohan@hnu.edu.cn.

Supplementary Note 1 | Characterisation of the light penetration depth.

Since layer thickness is a critical parameter in regulating printing quality, adjusting it to match the curing depth is essential to avoid the over-curing or incomplete-curing effects[1]. The former can reduce surface quality and pattern accuracy, while the latter can cause adhesion problems between adjacent layers. Jacob's working curve[2], describing the relation between curing depth and incident light energy, was established for PEG-GelMA/tartrazine and PEG-GelMA/Cur-Na bioink (see Supplementary Fig. 6) to determine the optimal printing parameters. The layer thickness (100 μm) was selected to achieve appropriate printing speed and vertical resolution. Based on the working curves, the light intensity and exposure time were then tuned to be 13 mW cm^{-2} and 13 s, respectively, ensuring that the curing depth for both bioink is slightly larger than the layer thickness necessary to enhance the bonding of two neighbouring layers.

Supplementary Note 2 | Characterisation of mechanical properties.

Compressive tests have been conducted to investigate the influence of Cur-Na on the mechanical properties of the generated hydrogel. The elastic modulus is lowered slightly by approximately 12% - 16%, indicating that the impact of Cur-Na and tartrazine on the mechanical properties is minor (Fig. 2c-d). It is likely due to the low molar absorptivity (ε = 50 M^{-1} cm^{-1} at 405 nm) of LAP, a photoinitiator employed in this study. Therefore, a small portion of photoabsorber (1.0 mM in this case) is enough to inhibit excessive light penetration[3].

Supplementary Note 3 | The lateral printing resolution analysis.

As shown in Fig. 4c, the printing resolution of the tartrazine-based bioink reduces when light energy increases, demonstrating strong sensitivity to light energy dosage. It is likely due to the enhanced scattering, and 1 mM tartrazine is insufficient to absorb the scattered light. We tried to increase the concentration of tartrazine (2-3 mM) to compensate for the increased scattering effect when the relative energy was amplified to $E_r = 2$. However, improvement in

print resolution was not achieved. In contrast, the unresolved area was enlarged when the concentration of tartrazine became larger (see Supplementary Fig. 8). This phenomenon can be explained by the gelation kinetics of polymerisation (see Fig. 2a). Increasing the amount of tartrazine reduces the curing depth and prolongs the gel time, leading to incomplete curing and the resulting deteriorated resolution. These results indicate that re-optimisation is always required when printing conditions change, and obtaining the most optimal printing accuracy is challenging using the purely photoabsorbing-based method.

Supplementary Note 4 | Printing performance of multi-layer fabrication.

To measure the blockage of the channels of the printed structures, the channels were cut across the central cross-section (Supplementary Fig. 11). In the case of pure PEG-GelMA bioink, all the channels were blocked. The shape of the cylinder cannot be maintained when the height of the cylindrical samples increases (see Supplementary Fig. 11a). For the PEG-GelMA/Tartrazine bioink, as the height increases to 3000 μm, the channels with smaller diameters of 300 μm and 500 μm are blocked (see Supplementary Fig. 11b) partially from the bottom of the fabricated parts. The PEG-GelMA/Cur-Na bioink demonstrates the superior capability for printing 3D channelled structures. All the channels are fabricated successfully regardless of the height of the printed structures (see Supplementary Fig. 11c).

Supplementary Note 5 | Imaging of generated hydrogels

One of the reasons for synthesising Cur-Na using sodium bicarbonate is to modify the hydrophilicity. Cur-Na has a water solubility of as large as 51 mg mL-1 and thus can be easily washed out of the fabricated hydrogel. We did not take any extra post-processing and did not encounter any difficulties with imaging (see the confocal images in Fig. 7). Another function of Cur-Na is to react with free radicals to hinder the solidification at regions illuminated by scattered light, therefore a tiny fraction of added Cur-Na will be crosslinked into hydrogels inevitably. However, a small amount of Cur-Na (1 mM) is adequate to confine the light-scattering effect. The printed structures are transparent enough for imaging after printing,

although a very light-yellow colour is virtually noticeable. The release experiment was conducted by immersing a pie-shaped sample in PBS solution (see Supplementary Fig. 15). The non-crosslinked Cur-Na was released within 4 hours, and a small fraction was encapsulated in the printed object, showing a pale-yellow colour.

Supplementary Figures

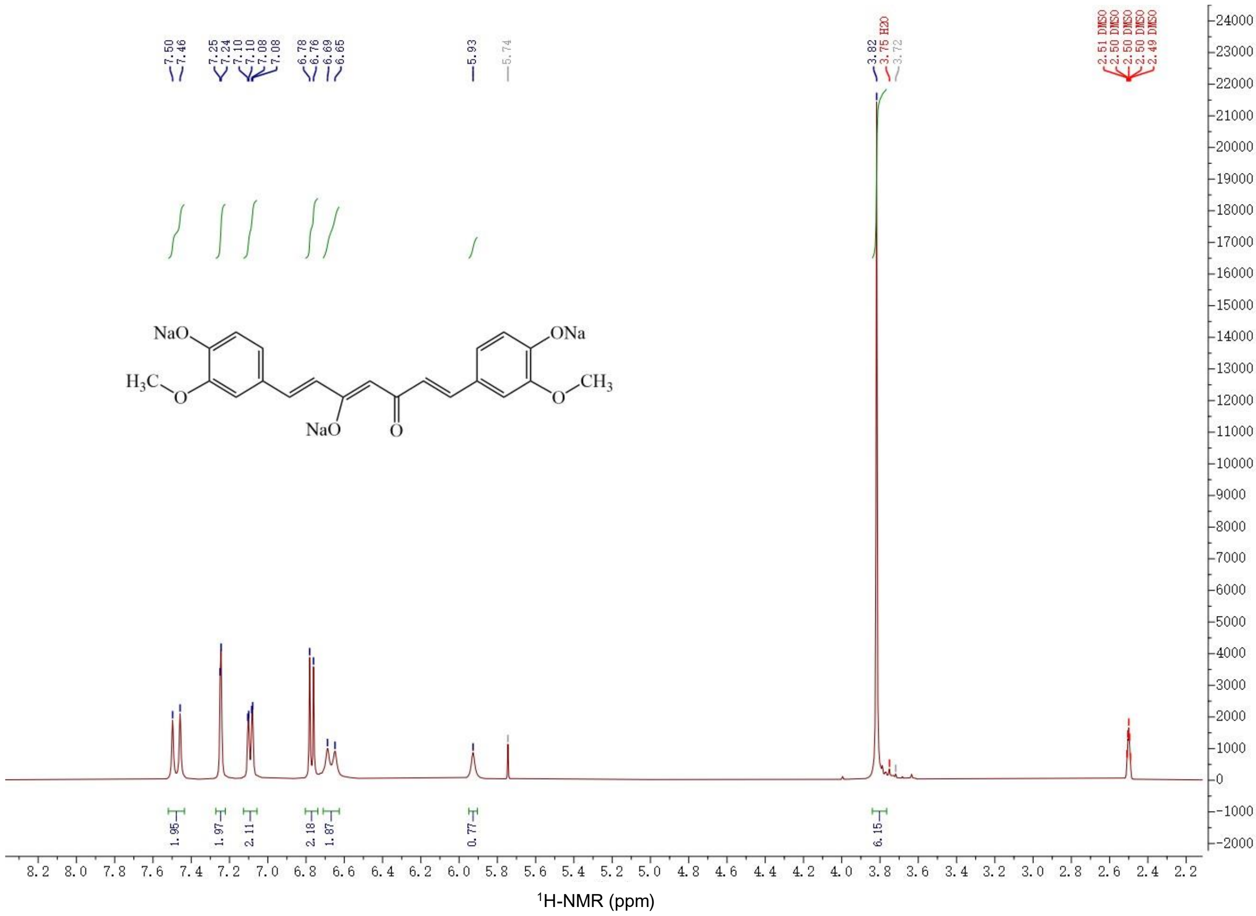

Supplementary Figure 1. ^{1}H-NMR spectrum of Cur-Na. ^{1}H-NMR (400 MHz, DMSO-d_6). δ (ppm) = 7.48 (d, J=15.7, 2H), 7.25 (d, J=2.0, 2H), 7.10 (dd, J=8.3, 2.0, 2H), 6.78 (d, J=8.1, 2H), 6.67 (d, J=15.8, 2H), 5.93 (s, 1H), 3.82 (s, 6H).

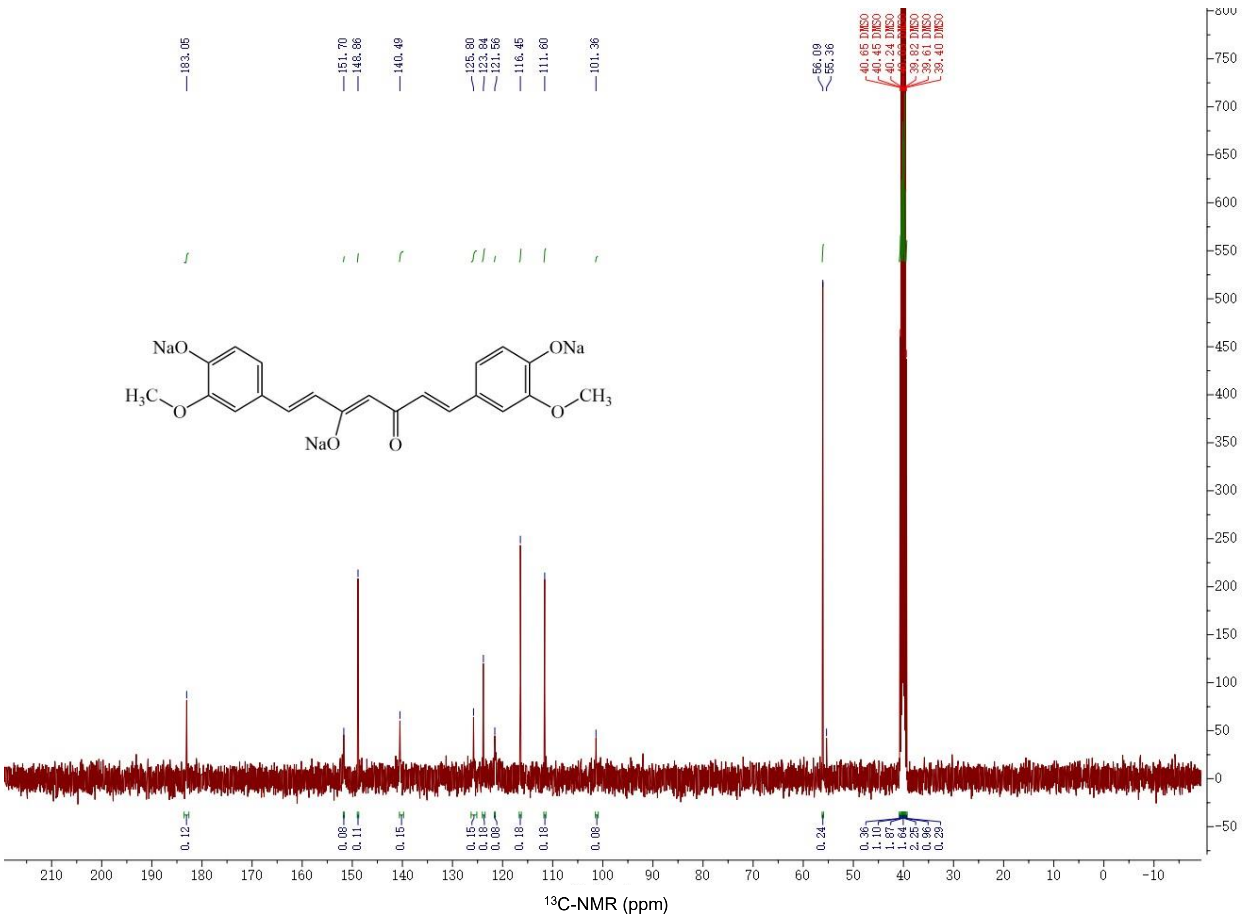

Supplementary Figure 2. ^{13}C-NMR spectrum of Cur-Na. ^{13}C-NMR (101 MHz, DMSO-d_6). δ (ppm): 183.05, 151.70, 148.86, 140.49, 125.80, 123.84, 121.56, 116.45, 111.6, 101.36, 56.09, 55.36.

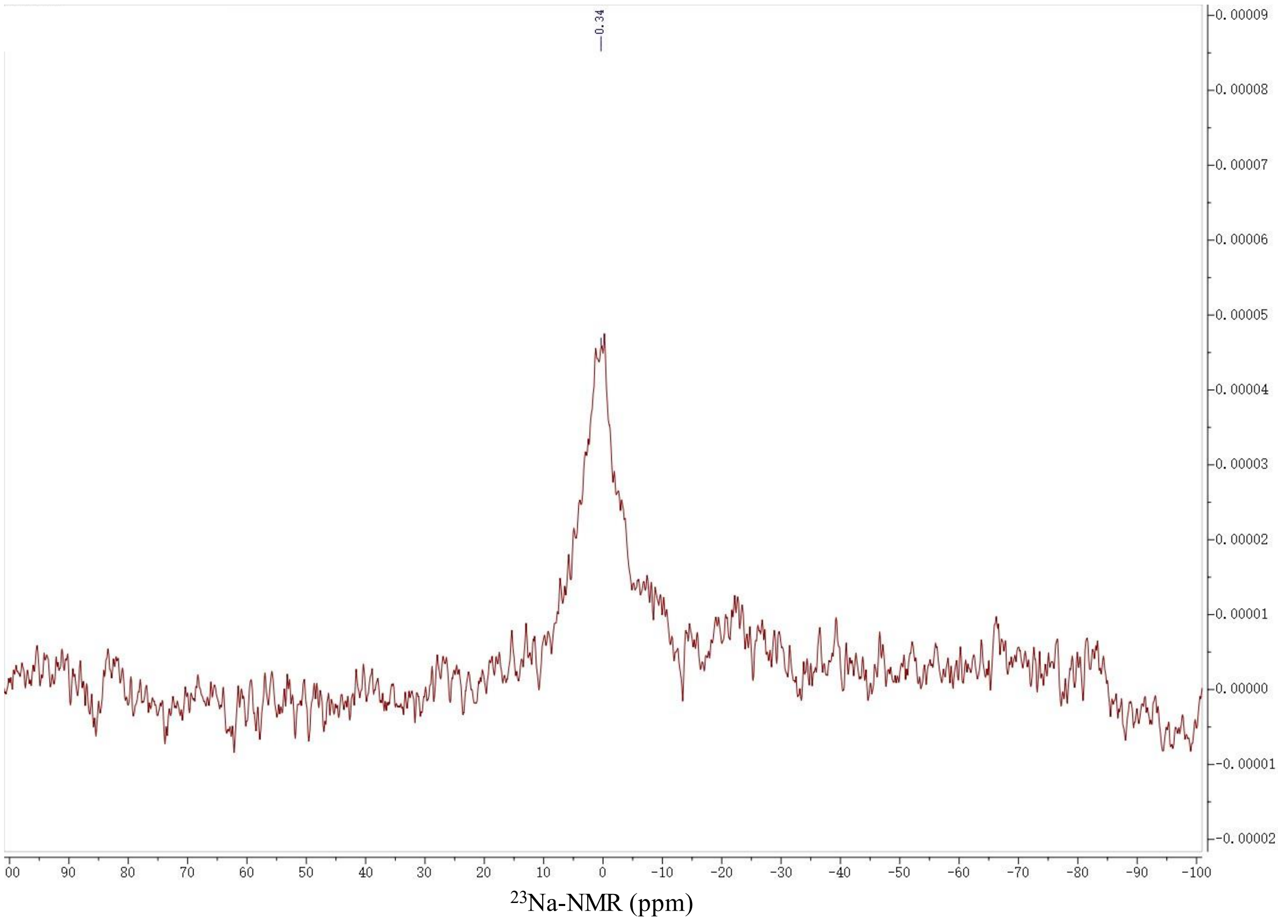

Supplementary Figure 3. ^{23}Na-NMR spectrum of Cur-Na. ^{23}Na-NMR (159 MHz, DMSO-d_6) δ (ppm): 0.34.

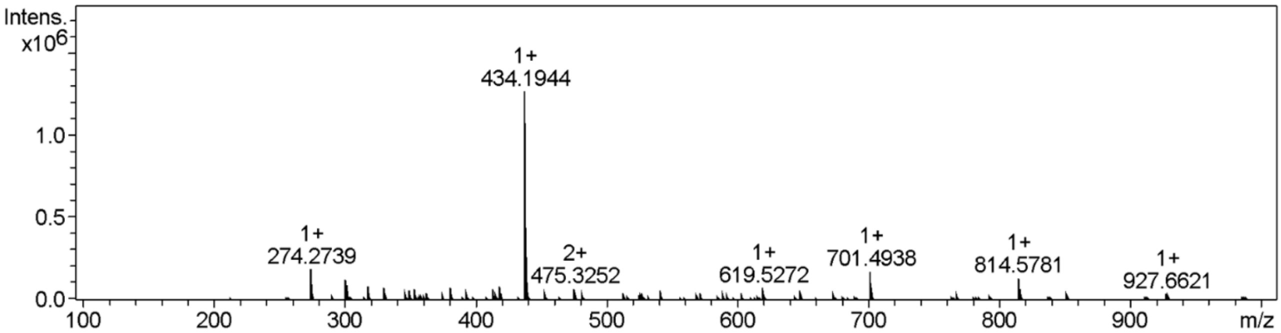

Supplementary Figure 4. High Resolution Mass Spectrometer (HRMS) analysis of Cur-Na. The molecular weight (434) matches that of the structural formula of Cur-Na.

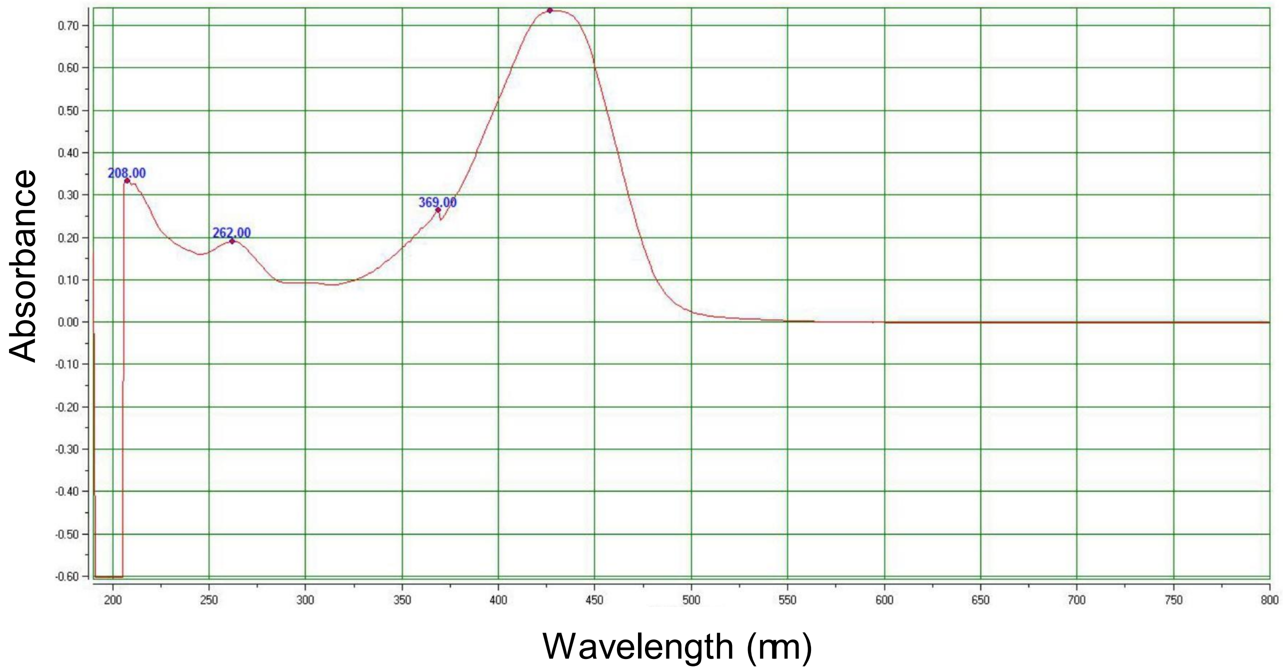

Supplementary Figure 5. UV-vis absorption spectrum of Cur-Na. The absorption peak of Cur-Na is approximately 425 nm, which is close to the wavelength of the light source of the printer (405 nm).

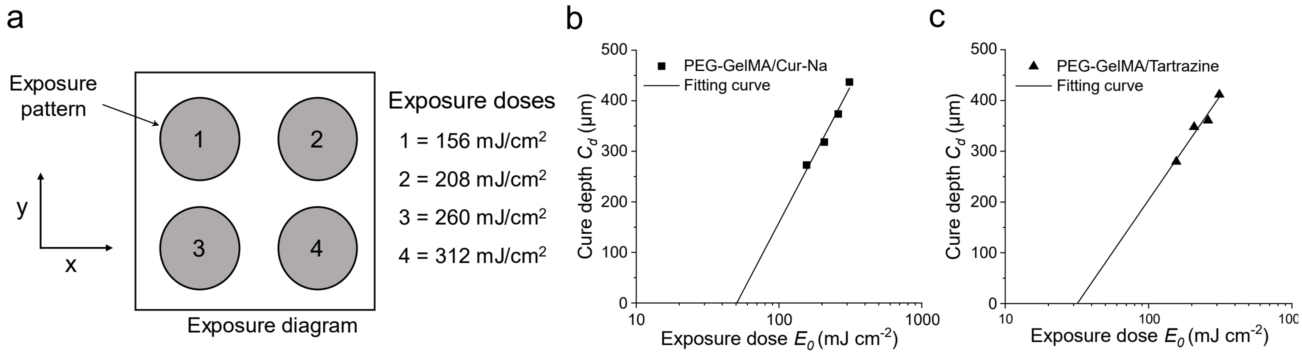

Supplementary Figure 6. Characterisation of the penetration depth. **(a)** Four circular specimens cured with different exposure doses varying between 156 and 312 mJ cm^{-2}. **(b)** The working curve for the PEG-GelMA/Cur-Na bioink, R^2 = 0.97358. (**c)** The working curve for the PEG-GelMA/Tartrazine bioink, R^2 = 0.95492.

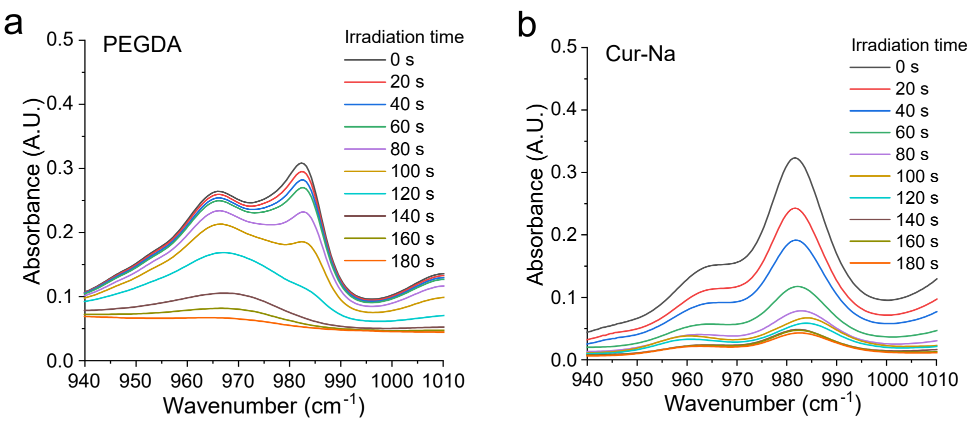

Supplementary Figure 7. FT-IR analysis for polymerising PEGDA and Cur-Na. Characteristic peak absorptions of C=C bond were recorded every 20 s from 0 s to 180 s for **(a)** PEGDA and **(b)** Cur-Na.

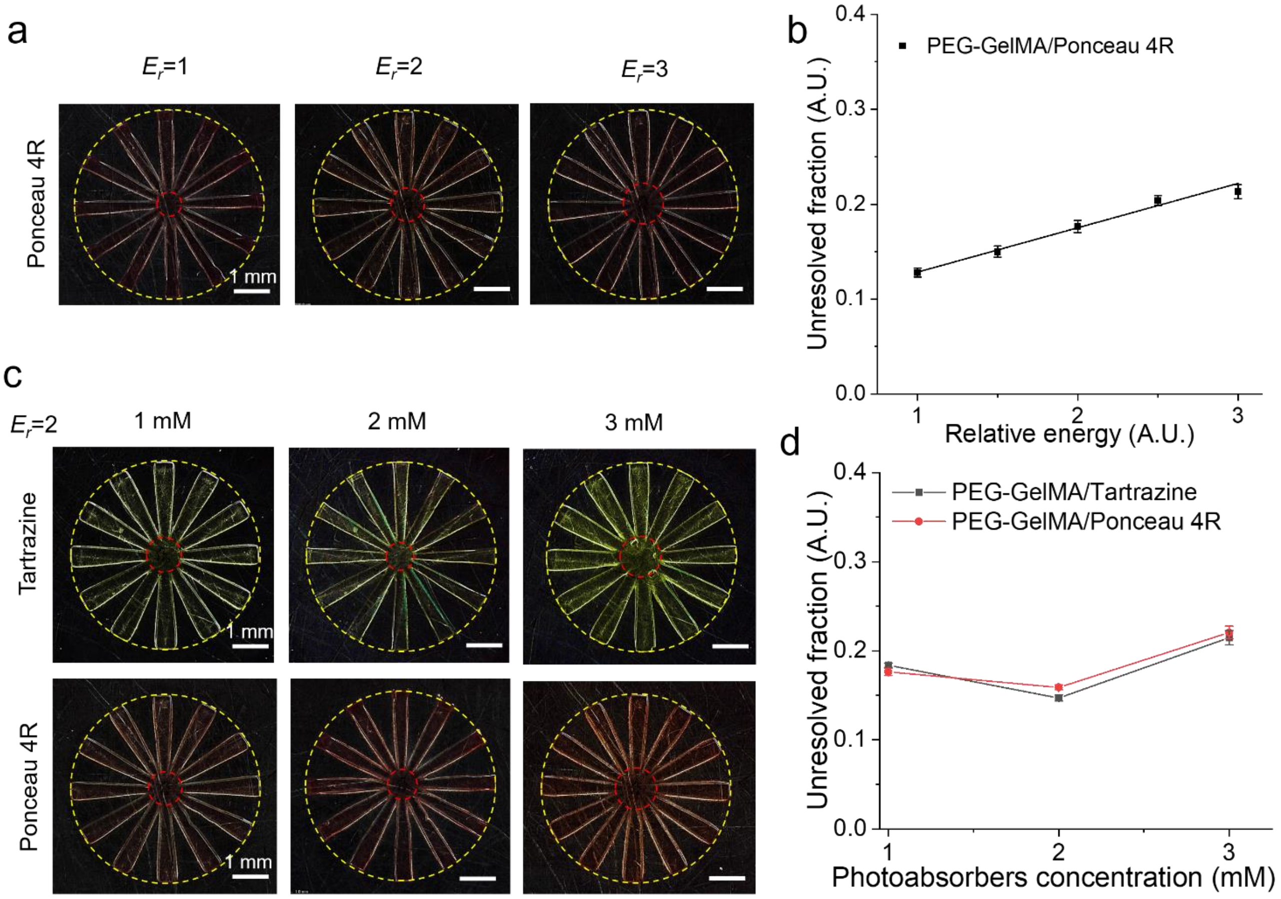

Supplementary Figure 8. The lateral printing resolution analysis for the PEG-GelMA/Tartrazzine and PEG-GelMA/Ponceau 4R bioinks. **(a)** Microscopic images of printed structures, showing the unresolved areas (red dashed circles) as a function of relative exposure energy for PEG-GelMA/Ponceau 4R bioink. **(b)** Quantitative relation of unresolved fraction against exposure energy. *n* = 3 independent samples. Data are presented as mean values ± standard deviation. **(c)** Microscopic images of printed structures, showing the unresolved areas (red dashed circles) as a function of the concentration of the photoabsorbers. **(d)** Quantitative relation of unresolved fraction against the concentration of photoabsorbers. When the light intensity is doubled, the amount of photoabsorbers required to absorb excessive light increases. *n* = 3 independent samples. Data are presented as mean values ± standard deviation. Scale bar: 1 mm.

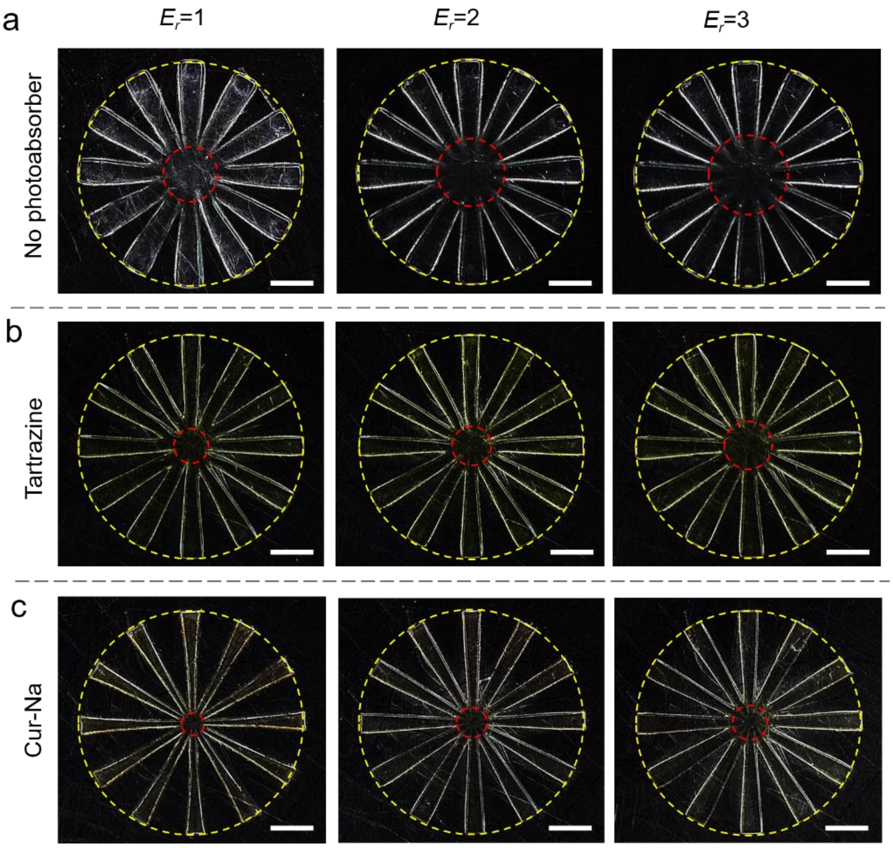

Supplementary Figure 9. Microscopic images of printed structures encapsulated with PC-12 cells, showing the unresolved areas (red dashed circles) as a function of relative exposure energy for pure **(a)** PEG-GelMA bioink and those added with **(b)** tartrazine and **(c)** Cur-Na. Images a-c are representatives of *n* = 3 independent experiments. Scale bar: 1 mm.

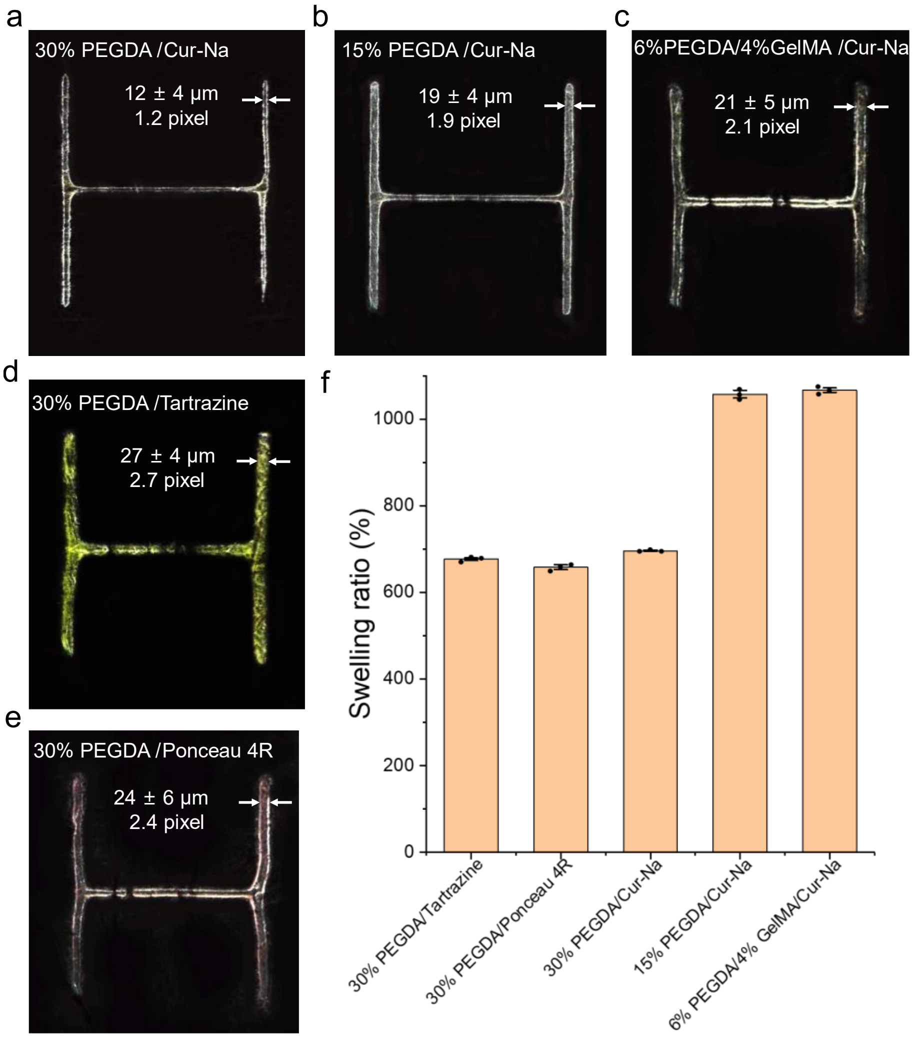

Supplementary Figure 10. Printing resolution tests for various hydrogels added with different photoinhibiting additives: **(a)** 30% PEGDA/CurNa, **(b)** 15% PEGDA/CurNa, **(c)** 6% PEGDA + 4% GelMA/CurNa, **(d)** 30% PEGDA/Tartrazine and **(e)** 30% PEGDA/Ponceau. Images a-e are representatives of *n* = 3 independent experiments. **(f)** Swelling ratio of the hydrogels. The linewidth of the H letter was designed to be 1 pixel (10 μm). *n* = 3 independent samples. Data are presented as mean values ± standard deviation.

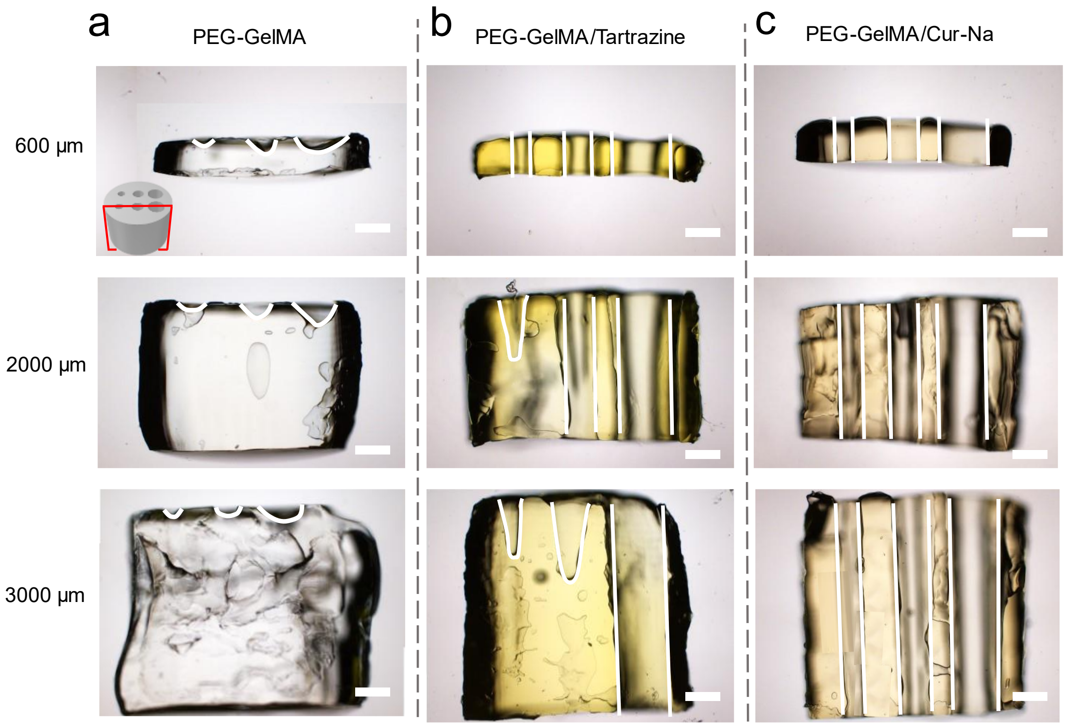

Supplementary Figure 11. Cross-section of the printed channels showing the variation of blockage with changes in the height of the cylindrical samples. **(a)** PEG-GelMA hydrogel. **(b)** PEG-GelMA/Tartrazine hydrogel. **(c)** PEG-GelMA/Cur-Na hydrogel. Scale bar: 500 µm. Images a-c are representatives of *n* = 3 independent experiments.

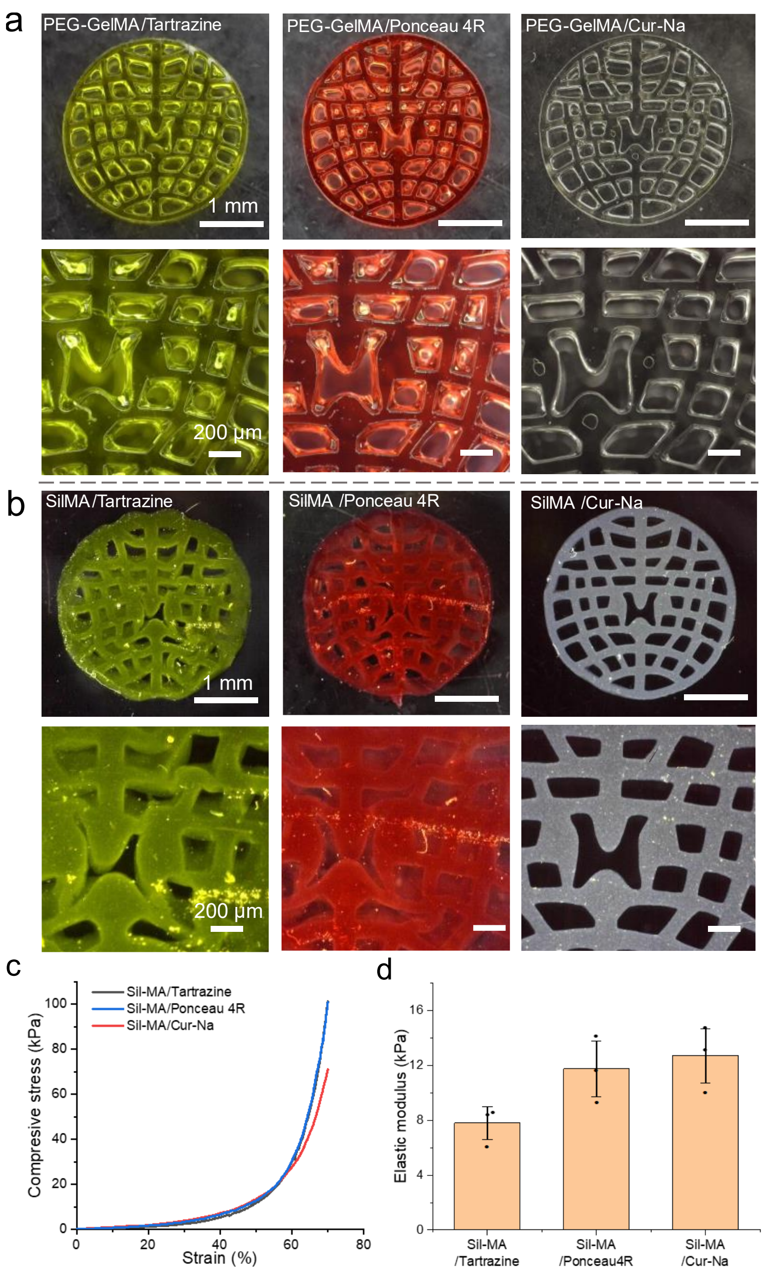

Supplementary Figure 12. Performance comparison of different hydrogels and photoinhibiting additives in printing the spinal cord scaffold designed based on micro-CT scanning data. **(a)** PEG-GelMA bioink. **(b)** Silk fibroin modified by glycidyl methacrylate (Sil-MA) bioink. Images a-b are representatives of *n* = 3 independent experiments. Note that part of the images are the same as that in Fig. 7a for the convenience of comparison. **(c)** Strain-stress curves and **(d)** Elastic modulus for the Sil-MA hydrogels. Cur-Na performed superbly in patterning 3D complex constructs featuring irregular channels and thin-walled networks, reproducing the designed fine features. *n* = 3 independent samples. Data are presented as mean values ± standard deviation. Each row of the images uses the same scale bar.

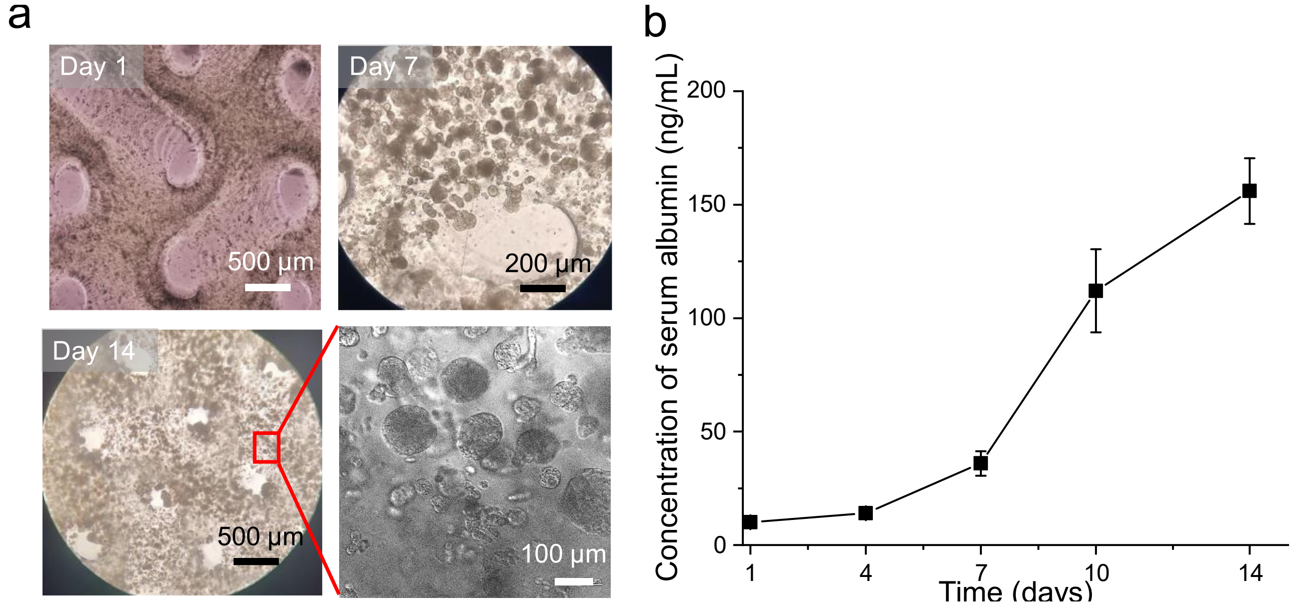

Supplementary Figure 13. Evaluation of hepatic function for the gyroid scaffold encapsulated with HepG2 cells. **(a)** Microscopic images showing the changes in cell growth. Sphere formation was observed on day 7. **(b)** The protein expression of albumin. *n* = 3 independent samples. Data are presented as mean values ± standard deviation.

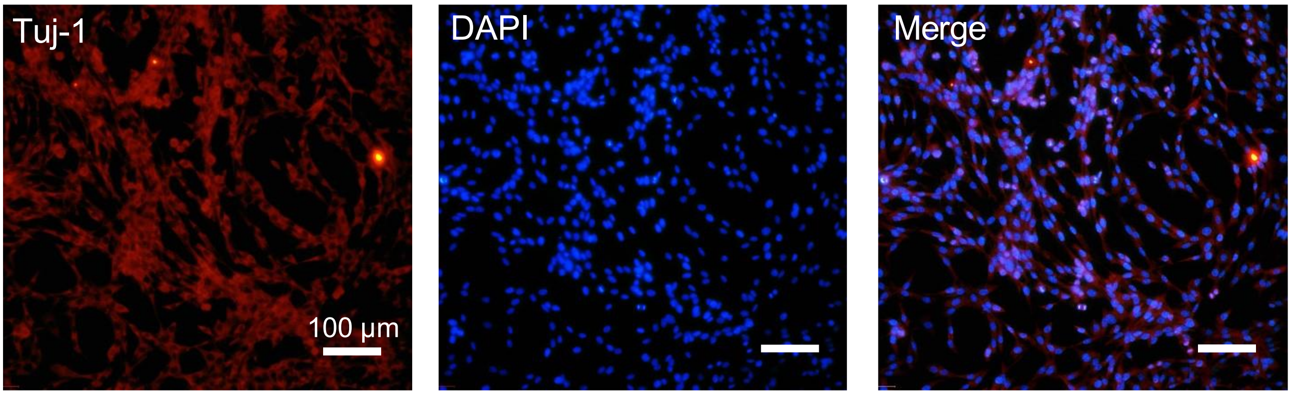

Supplementary Figure 14. In vitro PC-12 cells differentiation after being cultured 7 days in a differentiation medium containing 1 mM Cur-Na: Beta3-Tubulin (red) and cell nucleus (blue). Images are representatives of *n* = 3 independent experiments. Each row of the images uses the same scale bar.

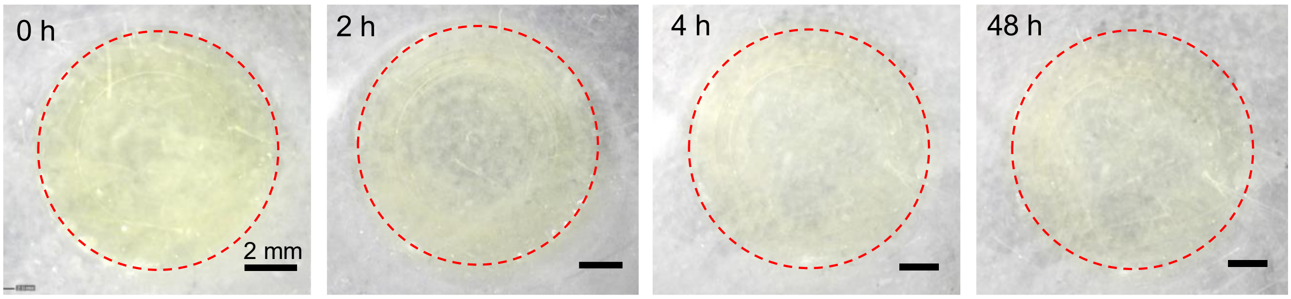

Supplementary Figure 15. Release experiment of Cur-Na conducted by immersing a pie-shaped sample in PBS solution. The non-crosslinked Cur-Na is released within 4 hours, and a small fraction is encapsulated in the printed object, showing a pale-yellow colour. Images are representatives of n = 3 independent experiments. Scale bar: 2 mm.

Supplementary Tables

Supplementary Table 1 | High-resolution mass spectral (HRMS) data

#	m/z	I	I %
1	274.2739	185348	14.7
2	301.1409	125658	9.9
3	302.3051	94503	7.5
4	318.2999	81664	6.5
5	330.3365	71944	5.7
6	349.1102	55312	4.4
7	353.2659	70204	5.6
8	362.2412	38783	3.1
9	381.2974	76207	6.0
10	413.2662	62502	4.9
11	418.7832	86225	6.8
12	419.2843	44392	3.5
13	434.1944	1263220	100.0
14	435.1971	309614	24.5
15	436.1994	52766	4.2
16	475.3252	66666	5.3
17	481.2618	36206	2.9
18	512.5036	44021	3.5
19	527.1609	41079	3.3
20	540.5347	55006	4.4
21	571.1631	41580	3.3
22	588.4091	36185	2.9
23	603.1680	41224	3.3
24	619.5272	72442	5.7
25	647.5581	55694	4.4
26	701.4938	173625	13.7
27	702.4964	71680	5.7
28	814.5781	133623	10.6
29	815.5809	64217	5.1
30	927.6621	37256	2.9

Supplementary References

1. Yu, K., et al. Printability during projection-based 3D bioprinting. *Bioact. Mater.* (2021).
2. Yu, C., et al. Photopolymerizable Biomaterials and Light-Based 3D Printing Strategies for Biomedical Applications. *Chem. Rev.* **120,** 10695-10743 (2020).
3. Grigoryan, B., et al. Multivascular networks and functional intravascular topologies within biocompatible hydrogels. Science 458-464 (2019).
