# Supplementary material for: Photoinhibiting via simultaneous photoabsorption and free-radical reaction for high-fidelity light-based bioprinting
Source: Nat Commun. 2023 May 27;14:3063. doi: 10.1038/s41467-023-38838-2 (PMC10224992; doi:10.1038/s41467-023-38838-2)
Supplement: Supplementary file 2 — Description of Additional Supplementary Files [file 41467_2023_38838_MOESM2_ESM.pdf]

Description of additional supplementary files

Supplementary Movie 1. Perfusion of the vascular scaffold (Fig. 5b) conducted via injection with a coloured dye solution.
