# Supplementary material for: Photoinhibiting via simultaneous photoabsorption and free-radical reaction for high-fidelity light-based bioprinting
Source: Nat Commun. 2023 May 27;14:3063. doi: 10.1038/s41467-023-38838-2 (PMC10224992; doi:10.1038/s41467-023-38838-2)
Supplement: Supplementary file 4 — Reporting Summary [file 41467_2023_38838_MOESM4_ESM.pdf]

nature portfolio

Corresponding author(s): Feng Chen; Xiaoxiao Han

Last updated by author(s): May 11, 2023

Reporting Summary

Nature Portfolio wishes to improve the reproducibility of the work that we publish. This form provides structure for consistency and transparency in reporting. For further information on Nature Portfolio policies, see our Editorial Policies and the Editorial Policy Checklist.

Statistics

For all statistical analyses, confirm that the following items are present in the figure legend, table legend, main text, or Methods section.

n/a	Confirmed	
☐	☒	The exact sample size (*n*) for each experimental group/condition, given as a discrete number and unit of measurement
☐	☒	A statement on whether measurements were taken from distinct samples or whether the same sample was measured repeatedly
☐	☒	The statistical test(s) used AND whether they are one- or two-sided<br>*Only common tests should be described solely by name; describe more complex techniques in the Methods section.*
☒	☐	A description of all covariates tested
☒	☐	A description of any assumptions or corrections, such as tests of normality and adjustment for multiple comparisons
☐	☒	A full description of the statistical parameters including central tendency (e.g. means) or other basic estimates (e.g. regression coefficient) AND variation (e.g. standard deviation) or associated estimates of uncertainty (e.g. confidence intervals)
☐	☒	For null hypothesis testing, the test statistic (e.g. *F*, *t*, *r*) with confidence intervals, effect sizes, degrees of freedom and *P* value noted<br>*Give P values as exact values whenever suitable.*
☒	☐	For Bayesian analysis, information on the choice of priors and Markov chain Monte Carlo settings
☒	☐	For hierarchical and complex designs, identification of the appropriate level for tests and full reporting of outcomes
☒	☐	Estimates of effect sizes (e.g. Cohen's *d*, Pearson's *r*), indicating how they were calculated

Our web collection on statistics for biologists contains articles on many of the points above.

Software and code

Policy information about availability of computer code

Data collection	Anton Par parallel plate rheometer was controlled by RheoCompass software.<br>Thermo Scientific FT-IR spectroscopy was controlled by Omnic software.<br>Thermo Scientific Multiskan FC was controlled by Skanlt RE 6.1.1 software.
Data analysis	The 3D model were designed using the Computer-Aided Design (CAD) 2021<br>The 3D model to be printed was sliced by using a BMF 3D slicer software.<br>Microscopy images were obtained using the Dino-Lite microscope with the DinoCapture 2.0 software.<br>Confocal images were obtained using the Nikon Ti-E+A1 MP microscope with the NIS-Elements Viewer 4.50 software.<br>Fluorescent images were obtained using the Olympus IX73 microscope with the cellSens Standard software.<br>Origin 2018 software was used for plotting.<br>Significant differences between the means of parameters were calculated using the SPSS 17.0 software.<br>ImageJ was used to measure the printed structure's dimensions.<br>The NMR data was analysed using MestReNova14 software.

For manuscripts utilizing custom algorithms or software that are central to the research but not yet described in published literature, software must be made available to editors and reviewers. We strongly encourage code deposition in a community repository (e.g. GitHub). See the Nature Portfolio guidelines for submitting code & software for further information.

Data

Policy information about availability of data

All manuscripts must include a data availability statement. This statement should provide the following information, where applicable:

- Accession codes, unique identifiers, or web links for publicly available datasets
- A description of any restrictions on data availability
- For clinical datasets or third party data, please ensure that the statement adheres to our policy

The data supporting the findings of this study are available in the paper and the Supplementary Information, and can be obtained upon request from the corresponding author fchen@hnu.edu.cn. Source data are provided together with this paper.

Human research participants

Policy information about studies involving human research participants and Sex and Gender in Research.

Reporting on sex and gender	Human research participants were not involved in this study.
Population characteristics	Human research participants were not involved in this study.
Recruitment	Human research participants were not involved in this study.
Ethics oversight	Human research participants were not involved in this study.

Note that full information on the approval of the study protocol must also be provided in the manuscript.

Field-specific reporting

Please select the one below that is the best fit for your research. If you are not sure, read the appropriate sections before making your selection.

[x] Life sciences [ ] Behavioural & social sciences [ ] Ecological, evolutionary & environmental sciences

For a reference copy of the document with all sections, see nature.com/documents/nr-reporting-summary-flat.pdf

Life sciences study design

All studies must disclose on these points even when the disclosure is negative.

Sample size	No statistical method was used to determine the sample size. Sample size was chosen based on previous experience and standards in the field. A minimum of three independent experiments for the majority of conditions. All sample sizes, statistical analysis, and P-value were indicated in the manuscript and figure legends. For in vitro experiments, cell culturing samples were analysed at various time points referring to "Kim, S. H., et al. Precisely printable and biocompatible silk fibroin bioink for digital light processing 3D printing. Nat. Commun. 9, 1620 (2018)". The geometric size of the spinal cord scaffold is determined referring to "Koffler, J., et al. Biomimetic 3D-printed scaffolds for spinal cord injury repair. Nat. Med. 25, 263-269 (2019)". The geometric size of the vascular scaffold is referred to as "Han, X., Bibb, R. & Harris, R. Engineering design of artificial vascular junctions for 3D printing. Biofabrication 8, 025018 (2016)".
Data exclusions	No data were excluded from the analysis.
Replication	All the printouts for each experiment are not less than three. 90% - 100% attempts were successful, depending on the geometric complexity, machine conditions and operational environment.
Randomization	The imaging of all printed and cultured samples was randomly allocated.
Blinding	The cell samples were allocated to hydrogel materials in a single-blind approach. The persons performing sample preparation were aware of the identity of hydrogel materials, but unaware of the identity of cell groups. All data have been documented and are available from the corresponding authors upon reasonable request.

Reporting for specific materials, systems and methods

We require information from authors about some types of materials, experimental systems and methods used in many studies. Here, indicate whether each material, system or method listed is relevant to your study. If you are not sure if a list item applies to your research, read the appropriate section before selecting a response.

Materials & experimental systems

n/a	Involved in the study
	[x] Antibodies
	[x] Eukaryotic cell lines
[x]	Palaeontology and archaeology
[x]	Animals and other organisms
[x]	Clinical data
[x]	Dual use research of concern

Methods

n/a	Involved in the study
[x]	ChIP-seq
[x]	Flow cytometry
[x]	MRI-based neuroimaging

Antibodies

Antibodies used

Anti-beta III tubulin (Tuj-1, 1:500, ab18207, Abcam).
Goat anti-rabbit 546 (1:500, a11305, Invitrogen).
Human Albumin ELISA KIT (1:1, SEKH-0081, Solarbio)
DAPI (1:1, GTX30920, GeneTex)

Validation

Anti-beta III tubulin (https://www.abcam.cn/products/primary-antibodies/beta-iii-tubulin-antibody-neuronal-marker-ab18207.html)
Goat anti-rabbit 546 (https://www.thermofisher.cn/cn/zh/antibody/product/Goat-anti-Rabbit-IgG-H-L-Highly-Cross-Adsorbed-Secondary-Antibody-Polyclonal/A-11035)
Human Albumin ELISA KIT (https://www.solarbio.com/goods.php?id=38429)
DAPI (https://www.genetex.cn/Product/Detail/Fluoroshield-trade-with-DAPI/GTX30920)

Eukaryotic cell lines

Policy information about cell lines and Sex and Gender in Research

Cell line source(s)

The PC-12 cells (from Procell Life Science&Technology Co., Ltd, China) separated from the pheochromocytoma of adrenal gland of SD rats were cultured and a cell line was established in vitro.
The HepG2 cells (from Procell Life Science&Technology Co., Ltd, China) were isolated from a hepatocellular carcinoma of a 15-year-old, White, male youth with liver cancer.

Authentication

The morphologies of PC-12, HepG2 were authenticated at the time of purchase.

Mycoplasma contamination

The cell line has been tested negative for mycoplasma contamination.

Commonly misidentified lines
(See ICLAC register)

The cell used in the research are not presented in the ICLAC register.
